# Emotional Eating Interventions for Adults Living With Overweight and Obesity: A Systematic Review and Meta‐Analysis of Behaviour Change Techniques

**DOI:** 10.1111/jhn.13410

**Published:** 2025-01-06

**Authors:** D. Power, A. Jones, C. Keyworth, P. Dhir, A. Griffiths, K. Shepherd, J. Smith, G. Traviss‐Turner, J. Matu, L. Ells

**Affiliations:** ^1^ School of Health, Obesity Institute Leeds Beckett University Leeds UK; ^2^ School of Psychology Liverpool John Moores University Liverpool UK; ^3^ School of Psychology University of Leeds Leeds UK; ^4^ School of Health and Life Sciences Teesside University Middlesbrough Tees Valley UK

**Keywords:** behaviour change technique, emotional eating, obesity, psychological intervention, systematic review, weight management

## Abstract

**Background:**

Emotional eating (EE) is a barrier to the long‐term success of weight loss interventions. Psychological interventions targeting EE have been shown to reduce EE scores and weight (kg), though the mechanisms remain unclear. This review and meta‐analysis aimed to identify the specific behaviour change techniques (BCTs) associated with improved outcomes.

**Methods:**

This is a review update and extension, with new studies extracted from searches of CINAHL, PsycINFO, MEDLINE and EMBASE 1 January 2022 to 31 April 2023. EE interventions for adults with BMI > 25 kg/m^2^ were considered for inclusion. Paper screening, extraction, BCT‐coding and risk of bias were completed using the Template for Intervention Description and Replication (TIDieR) checklist, Behaviour Change Taxonomy v1 (BCTTv1) and Risk of Bias2 (RoB2)/Risk of Bias In Non‐randomised Studies (ROBINS‐I) tool. Narrative syntheses and random effects multi‐level meta‐analyses were conducted.

**Results:**

In total, 6729 participants were included across 47 studies (13 identified in the update). Forty‐two studies contributed to the pooled estimate for the impact of interventions on EE (SMD = −0.99 [95% CI: −0.73 to −1.25], *p* < 0.001). Thirty‐two studies contributed to the pooled estimate for the impact of interventions on weight (−4.09 kg [95% CI: −2.76 to −5.43 kg], *p* < 0.001). Five BCTs related to identity, values and self‐regulation were associated with notable improvements to both weight and EE (‘incompatible beliefs’, ‘goal setting outcome’. ‘review outcome goals’, ‘feedback on behaviour’ and ‘pros/cons’).

**Conclusion:**

Implementation and evaluation of the highlighted BCTs are required. Weight management services should consider screening patients for EE to tailor interventions to individual needs.

## Summary

- Psychological interventions for emotional eating (EE) in individuals with overweight and obesity lead to significant reductions in both EE and weight.
- This review has identified key behaviour change techniques (BCTs) associated with the greatest improvements in weight and EE outcomes.
- Interventions emphasising values, future outcomes, self‐regulation, psychological flexibility and self‐compassion are likely to be most effective, but further testing of these BCTs is needed to strengthen the evidence.

**Abbreviations**
ACT: Acceptance and Commitment Therapy
BCT: behaviour change technique
BCTO: behaviour change technique ontology
BCTTv1: behaviour change taxonomy v1
CBT: cognitive behavioural therapy
EE: emotional eating
GRADE: Grading of Recommendations Assessment, Development and Evaluation
ME: mindful eating
PRISMA: Preferred Reporting Items for Systematic Reviews and Meta‐analyses
PTSD: post traumatic stress disorder
QCA: qualitative comparative analysis
RCT: randomised control trial
RIM: reflective‐impulsive model
RoB2: risk of bias 2
ROBINS‐I: Risk Of Bias In Non‐randomised Studies ‐ of Interventions
TIDieR: Template for Intervention Description and Replication
TTB: trauma‐informed theory of behaviour

## 1 Introduction

More than a billion people globally are living with obesity [1]. Overweight and obesity are associated with increased morbidity and mortality as well as reduced quality of life [2]. There has been limited success in treating and preventing obesity [3] with interventions often resulting in initial weight loss, followed by weight regain [4, 5, 6]. Causes of obesity are multifactorial, including socioeconomic, environmental, biological and psychological drivers [7]. In behavioural weight loss (BWL) interventions, 5%–10% weight loss targets are promoted due to associated health benefits [8]. Therefore many BWL interventions focus predominantly on attempts to address energy imbalances in the diet without adequately addressing psychological drivers of eating behaviour [9]. Both qualitative [10] and quantitative [11] research suggest emotional eating (EE) is a barrier to the long‐term success of weight loss interventions, and when treated effectively can lead to improved outcomes [12].

There is no ubiquitous definition of EE; however, Smith et al. (2023) define EE as “the tendency to eat energy dense and palatable foods, in response to negative emotions … including symptoms of anxiety and depression, negative self‐concept, overeating” (p. 3) [13]. There are several theories to explain how EE develops [14], including using it as a strategy to regulate internal processes, which may have roots in childhood trauma [15]. This is supported by evidence that EE is associated with emotional regulation difficulties [16], addiction [17] and post‐traumatic stress disorder (PTSD) [18]. Other theories have suggested that positive emotions may also lead to EE but appear to work by a different construct. Positive emotions are associated with unhealthy snacking [19], but are less likely to induce overeating [20]. These have not been explored in this review.

Although EE and binge eating are positively related [21, 22], EE is considered subclinical [23] and therefore people who experience it are often ineligible for treatment. Up to 58% of adults referred to weight management settings report experiencing EE [24], which is associated with a range of physical comorbidities, such as heart disease and diabetes [11], and psychological comorbidities such as depression [25]. Given this additional mental and physical burden associated with EE, effective treatment options are urgently required. Furthermore, without adequate support, people affected by EE are likely to engage in repeated attempts of restrictive dieting, which conversely is associated with further maladaptive eating behaviours [26].

Currently, cognitive behavioural therapy (CBT) is the gold standard psychological intervention for the treatment of obesity [27] and has been found to reduce EE symptoms [13, 28]. However, due to the intensity of CBT and the training required, interest has grown in third‐wave CBT interventions, such as Acceptance and Commitment Therapy (ACT), mindfulness and compassion‐focused therapy. Recent systematic reviews suggest that these interventions can reduce EE [29, 30] with conflicting evidence as to which treatment is superior.

A recent systematic review [13] of psychological interventions targeting EE amongst adults living with overweight or obesity, found significant, albeit small, reductions in weight (−1.08%, 95% CI: −1.66 to −0.49) and EE (−2.37%, 95% CI: −3.76 to −0.99) following treatment. Subgroup analysis showed that CBT was superior in reducing EE scores (−38%), followed by acceptance‐based interventions (−25%). However, it remains unclear as to which components or behaviour change techniques (BCTs) are effective. BCTs are the active components of behaviour change interventions that are tangible, reproducible and can facilitate behaviour change [31]. Identification of effective BCTs in reducing EE is crucial to successful EE intervention development. In line with the emotional‐regulation approach outlined above, BCTs that have shown to be effective in treating trauma (e.g., those which emphasise identity [32] and self‐regulation [33]) were hypothesised to be effective in addressing EE. These BCTs are postulated to induce positive behaviour change through their impact on the reflective‐impulsive model (RIM) and the trauma‐informed theory of behaviour (TTB).

Therefore, this review will provide an update and extension (BCT extraction) of the Smith et al. (2023) [13] review. This review is pivotal as BCTs have not yet been examined in relation to EE and will offer novel and evidence‐based recommendations for clinical practice.

The primary objective is to examine BCTs in effective EE interventions for adults living with overweight and obesity, measuring whether the number of BCTs included in an intervention is associated with reductions in weight and EE, and examining which BCTs are associated with the greatest reduction in weight and EE scores.

## 2 Methods

### 2.1 Protocol and Registration

This systematic review was prospectively registered with the International Prospective Register of Systematic Reviews (PROSPERO registration number CRD42023413966) and followed the Preferred Reporting Items for Systematic Reviews and Meta‐Analyses (PRISMA) guidelines and the PRISMA 2020 checklist [34]: Checklist reported in Supporting Information S1: Table S1.

### 2.2 Eligibility Criteria

As this review is an update and extension, the eligibility criteria used by Smith et al. [13] have been employed.

#### 2.2.1 Population

Participants were adults aged ≥ 18 years, of any sex, living in any country with a BMI > 25 kg/m^2^. Studies with < 70% of the sample with a BMI of ≥ 25 kg/m^2^ were excluded.

#### 2.2.2 Intervention

Included studies were published studies that evaluated psychological interventions, which had an EE component, aimed at adults living with overweight and obesity. Studies involving medical interventions or medical devices, post‐bariatric surgery and psychological therapies for weight loss that do not address EE were excluded.

#### 2.2.3 Comparator

The review was not limited to studies that included a comparator group.

#### 2.2.4 Outcomes

Primary outcomes were changes in weight (kg) and EE scores using validated emotional eating questionnaires. Secondary outcomes were other measures of health where this had been recorded (such as blood glucose, blood pressure and cholesterol).

#### 2.2.5 Study Design

Any primary published research that reported pre‐ and post‐intervention data was included. Animal studies, letters to editors and commentaries were excluded.

### 2.3 Search Strategy

A systematic literature search was conducted by two authors (D.P. and J.S.), which followed the same strategy as Smith et al. [13], who completed their search from inception to January 2022. We included their papers, and updated their search, using the same terms in the same databases to identify literature published after 1 January 2022, through to 31 April 2023. All intervention studies, which included a psychological component targeting EE for adults living with overweight and obesity, were considered for inclusion. D.P. searched CINAHL, PsycINFO and MEDLINE, and J.S. searched EMBASE. Appropriate protocol papers were identified, and authors were contacted where further information was needed. Studies were limited to the English language with no restrictions on geographical location. The search comprised of the following key terms: ‘Mindful*’, ‘Mindful eat*’, ‘Emotional Eating’, ‘Cognitive behavio*’, ‘Behavio* change’, ‘binge eat*’, ‘comfort eat*’, ‘self‐help’, ‘food addiction’, ‘acceptance and commitment therapy’, ‘ACT’, ‘intervention’, ‘treatment’. Full search description is provided in Supporting Information S1: 2.0 Search Strategy.

#### 2.3.1 Screening Process

**Figure 1:**
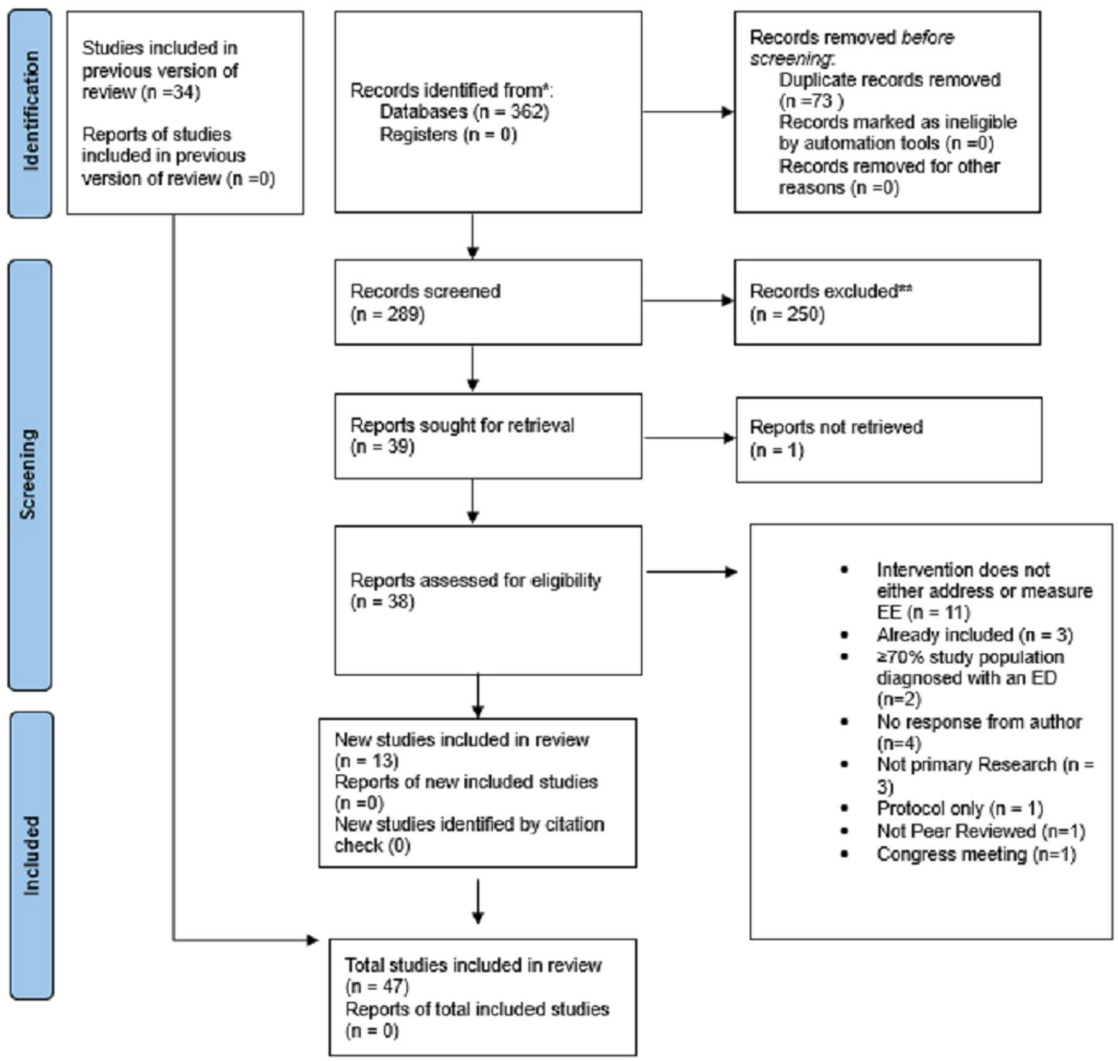
PRISMA flow chart of study selection.

All study titles were screened independently and in duplicate by two authors (D.P. and P.D.) who then met to check the agreement. Abstracts were retrieved and reviewed independently and in duplicate for studies, which required further information, and authors agreed on which titles to progress to full‐text review (see Figure 1). Full texts were reviewed by D.P. and A.G. in duplicate, any ambiguity was discussed, and eligible studies were confirmed. A third author was used for dispute resolution if necessary. A PRISMA flow diagram is provided to show the number of studies that were included at each stage and justification provided (Figure 1). Reasons for excluding studies which progressed to full paper review are provided in Supporting Information S1: Table S2.

### 2.4 Data Extraction

The data extraction template from Smith et al. (2023) [13] was used and extended to extract the same data fields from the included studies from the updated search. This included key information regarding participant characteristics, study design, sample size, mean age, study population, length of intervention, theoretical underpinnings, length of follow‐up and main findings, which were extracted by one author (D.P.) and used to develop Table 1. A second data extraction template was created and populated by one author (D.P.), according to the Template for Intervention Description and Replication (TIDieR) checklist [35], to retrieve more detailed information on each included study from across the original and updated search. This second TIDieR data extraction form was checked for agreement by a second author (K.S.). A high level of agreement, in terms of data extraction and interpretation of study details, was achieved (> 90%), with K.S. adding additional information to approximately 5% of the data extraction form. BCTs according to the Behaviour Change Taxonomy v1 (BCTTv1) [36] were extracted from each paper by one author (D.P.) and checked for agreement with author (C.K.). Both authors had completed online training in applying the taxonomy (bct-taxonomy.com). Good agreement was achieved (> 90%), where there was ambiguity, it was resolved through discussion.

**Table 1:** Summary of included studies.

Study	Sample	Interventions	No. of BCTs	Results
Afari et al. (2019) [37]	*N* = 88 (age: 57.3 years, 76.1% male)	90 days RCT including ACT and BWL	16	EE: %:↔ weight:↔
Ahern et al. (2022) [38]	*N* = 61 (age: 48 years, 84% female)	12 weeks RCT including online GSH based on ACT	27	EE:↓ weight: ↓
Annesi and Eberly (2023) [39]	*N* = 121 (100% female)	24 weeks of community‐based intervention involving self‐regulatory skills	10	EE:↓ weight:*
Annesi et al. (2016) [40]	*N* = 103 (age: 47.8 years, 100% female)	24 weeks RCT including CBT and BWL	10	EE:↓ weight: ↓
Annesi (2019) [41]	*N* = 152 (age: 48.6 years, 100% female)	28–99 weeks RCT including CBT and BWL	10	EE:↓ weight:*
Bacon et al. (2005) [42]	*N* = 35 (age: 40.4 years, 100% female)	24 weeks RCT including HAES and ABT	8	EE:↓ weight: ↔
Berman et al. (2022) [43]	*N* = 19 (age: 51 years, 100% female)	11 weeks RCT including HAES + ACT	11	EE:↓ weight:*
Braden et al. (2022) [44]	*N* = 39 (Age: 49.2 years, 100% female)	16‐week pilot trial of DBT and BWL	16	EE: ↓ weight:*
Carbine et al. (2022) [45]	*N* = 100 (age: 28.0 years, 53% female)	4‐week RCT including food specific ICT	1	EE:** weight: ↔
Carpenter et al. (2019) [46]	*N* = 75 (age: 47.3 years, 92% female)	24‐week RCT including mindfulness and BWL	7	EE: ↓ Weight: ↓
Chung et al. (2016) [47]	*N* = 22 (age: 50.1 years, 100% female)	24‐week intervention involving ME	4	EE: ↓ weight: ↔
Daubenmier et al. (2016) [48]	*N* = 194 (age: 47.5 years, 80% female)	5.5 month RCT involving mindfulness and BWL (including diet and exercise)	16	EE: ↓ weight:↓
Fang et al. (2023) [49]	*N* = 20 (age: 42.0 years, 75% female)	4‐week RCT including app‐based BWL support	0	EE: ↔ weight:**
Forman et al. (2013) [50]	*N* = 128 (age: 45.7 years)	40‐week RCT including acceptance strategies and BWL	14	EE:** weight:*
Frayn et al. (2020) [51]	*N* = 32 (46.7 years, 87.5% female)	1‐day single intervention group involving ACT	14	EE: ↓ weight: *
Geniş et al. (2022) [52]	*N* = 40 (41.7 years, 91.4% female)	8‐week pilot of CBT	7	EE: ↓ weight: ↓
Goldbacher et al. (2016)[53]	*N* = 79 (age: 45.6 years, 95% female)	20‐week RCT including BWL and EBT	13	EE:** weight: ↓
Hanson et al. (2018) [54]	*N* = 53 (age: 44.4 years, 78.8% female)	8‐week single group mindfulness study	12	EE: ↔ weight: ↔
Hanson et al. (2022) [55]	*N* = 289 (age 46.9 years, 74.4% female)	8‐week mindfulness intervention	12	EE:* weight: ↓
Hawkins et al. (2021) [56]	*N* = 48 (age: 43.6 years, 85% female)	23‐week group intervention including ABT	9	EE: ↓ weight: ↓
Hepdurgun et al. (2020)[57]	*N* = 51 (age: 40.1 years)	8‐week RCT including BWL	9	EE:* Weight: **
Hunot‐Alexander et al. (2021) [58]	*N* = 37 (age: 48.3 years, 93.8% female)	8‐week single group intervention including ATTI	10	EE: * weight: **
Kearney et al. (2012) [59]	*N* = 48 (Age: 49 years, 87.5% male)	8‐week single group intervention including MBSR	6	EE: ↔ Weight: ↔
Keränen et al. (2009) [60]	*N* = 20 (age: 52 years 25% male)	20‐week RCT including intensive counselling	10	EE: * weight: ↓
Kidd et al. (2013) [61]	*N* = 12 (age: 51.8, 100% female)	8‐week single group intervention including ME	8	EE: ↔ Weight: ↔
Kim et al. (2021) [62]	*N* = 583 (age: 53.7 years, 61.6% female)	24‐week RCT including a focus on dietary attitudes and BWL	6	EE: * Weight: ↔
Lillis et al. (2016) [63]	*N* = 162 (age: 50.2, 85% female)	24‐month RCT including BWL and ABT	18	EE: ↓ weight: ↓
Malkina‐Pykh (2012) [64]	*N* = 104 (age: 37.6 years, 69% female)	48‐week RCT including CBT and RMT	4	EE: ↓ weight: *
Manchón et al. (2022) [65]	*N* = 23 (age: 44.1 years, 100% female)	10‐week single group intervention study including ACT and BWL	11	EE: ↓ weight:*
Manzoni et al. (2009) [66]	*N* = 40 (100% female)	5‐week RCT of relaxation training	8	EE: ** weight: **
Mason et al. (2018) [67]	*N* = 104 (age: 46.07)	28‐day mindfulness phone‐based intervention	13	EE: ↓ weight: ↔
Mohseni et al. (2022) [68]	*N* = 96 (age: 42 years, 76% female)	1.5‐year single‐group pilot intervention of lifestyle and CBT	9	EE: ↓ weight: ↓
Moraes et al. (2021) [69]	*N* = 64 (age: 36.2 years, 77.4% female)	30‐week RCT involving BWL and CBT	14	EE: ↓ weight:↔
Mueller et al. (2023) [70]	*N* = 192 (age:50.7 years, 78.1% female)	12‐week RCT including online GSH based on ACT	27	EE: ↓ weight: ↔
Niemeier et al. (2012) [71]	*N* = 21 (age: 52.2 years, 90.5% female)	24‐week single group study design based on ABT and BWL	15	EE: ↓ weight: ↓
Paans et al. (2020) [72]	*N* = 372 (age: 47.8 years, 78.2% female)	1‐year RCT including FBA	13	EE: ↓ weight: ↔
Palmeira et al. (2017) [73]	*N* = 27 (age 42.0 years, 100% female)	12‐week RCT including ACT	11	EE: ↓ weight *
Paul et al (2022) [74]	*N* = 130 (age: 41.4 years, 74.6% female)	12‐week RCT including CBT	13	EE: ↓ weight:*
Rieger et al. (2017) [75]	*N* = 201 (age: 47.0 years, gender 73.6% female	1‐year RCT including CBT and support person	19	EE: ↓ weight: ↓
Roosen et al. (2012) [76]	N = 35 (Age: 39.2 years, 86% female)	20‐week single group design intervention study based on DBT	6	EE: ↓ weight:*
Salvo et al. (2022) [77]	*N* = 20 (age: 48.2 years, 100% female)	13‐week single‐group design intervention study based on MB‐EAT	10	EE: ↓ weight: ↔
Salvo et al. (2022) [78]	*N* = 284 (age: 40.4 years, 100% female)	10‐week RCT of ME	7	EE: ↓ weight: ↓
Sampaio et al. (2021) [79]	*N* = 27 (age: 49 years, 100% female)	7‐month RCT of meditative practice and mindfulness	6	EE: ↓ weight:*
Spadaro et al. (2017) [80]	*N* = 46 (age: 45.2 years, 87% female)	24‐week RCT including BWL and mindfulness	12	EE: ↓ weight: ↓
Tham and Chong (2020)[81]	*N* = 120 (57.5% female)	26‐week single group design involving CBT intervention	5	EE: ↓ weight: ↓
Thomas et al. (2019) [82]	*N* = 51 (age: 57.9 years, 100% female)	10‐week RCT including BWL and mindfulness	8	EE: ↓ weight:↔
Van Uytsel (2022) [83]	*N* = 1075 (age; 31.2, 100% female)	RCT including postpartum BWL advice and MI techniques	9	EE: ↔ Weight: ↓

Abbreviations: ABT, acceptance‐based therapy; ACT, acceptance and commitment therapy; ATTI, appetitive tailored trait intervention; BWL, behavioural weight loss; CBT, cognitive behavioural therapy; DBT, dialectical behavioural therapy; EBT, enhanced behavioural therapy; EE, emotional eating; FBA, food‐based activation; GSH, guided self‐help; HAES, health at every size; ICT, inhibitory control training; ME, mindful eating; MBEAT, mindfulness‐based eating awareness training; MBSR, mindfulness‐based stress reduction; MI, motivational interviewing; RMT, rhythmic movement therapy

* Results not reported.

** Unable to include data in the analysis due to missing components or how reported in the study.

↓ indicates a statistically significant reduction in outcome measure, 95% CI does not cross the line of no effect.

↔ no significant change, 95% CI crosses the line of no effect.

↑indicates a statistically significant increase in outcome measure.

EE is measured as a change in SMD score, weight is measured as a change in kg.

### 2.5 Risk of Bias

Risk of bias was assessed using the Risk of Bias 2 (RoB2) and Risk of Bias in Non‐randomised Studies—of Interventions (ROBINS‐I) tool for RCTS and non‐randomised studies respectively. Risk of bias was undertaken by one author (D.P.) and checked for agreement by a second author (A.G). The full list is provided in Supporting Information S1: Tables S6 and S7.

### 2.6 Data Synthesis

To be included in the meta‐analysis, complete data were required. Where data were incomplete, we contacted the original authors to request the missing data. For the meta‐analysis, we used a random effects model with restricted maximum likelihood estimators. For effects on weight loss within the intervention group, we calculated mean weight change (in kg), and for EE we calculated standardised mean differences (as different scales were used across different studies), using the ‘escalc’ function from the metafor R package [84]. In both cases, as the estimates were pre–post we imputed a correlation between each of 0.7, in line with Hofmann et al [85]. If weight was provided in a different unit (pounds, stones) we converted it to kg. To examine heterogeneity, we provided the *I* ^2^ index [86] in which 50% is indicative of moderate, and 75% is indicative of substantial heterogeneity. We also provide tau^2^ (*τ*²), which is the squared standard deviation of the effect sizes. For the examination of potential publication bias, we report Trim and Fill [87] and Egger's regression test [88]. To examine post‐test differences in intervention versus control groups we followed the same strategy as above; however, effect sizes did not require adjustment via coefficients.

In meta‐regressions, we examined whether the number of BCTs included in an intervention was associated with weight change or EE change. To examine the impact of individual BCT, we computed estimated effect sizes across studies where a BCT was identified. We then plotted each of these estimated effect sizes in a forest plot.

In moderation analyses, we examined delivery (in‐person vs. remote), format (group vs. individual vs. mixed), type of study (RCT or quasi‐experimental), length of intervention (in weeks) and quality of the study on weight change and EE. Analysis scripts are available here: https://osf.io/6bfdj/.

## 3 Results

### 3.1 Included Studies

All 34 papers from Smith et al. (2023) were included in this updated review accounting for studies published up until January 2022. Database searches from 1 January 2022 until 31 April 2023 returned 362 publications, 73 duplicates were removed and 289 abstracts were screened. Of these, 250 publications did not meet the inclusion criteria and out of the remaining 39 publications, 38 full texts were successfully retrieved and assessed for eligibility. Thirteen new studies were included in this updated review. Reasons for exclusions at the full‐text screening stage are summarised in the PRISMA flowchart in Figure 1 and Supporting Information S1 (Table S2). One of the included papers [38] is a protocol and has not yet been peer‐reviewed; however, we successfully contacted the authors for relevant data and permission to include it in this systematic review and meta‐analysis. One study [48] reported their EE data in a separate paper [89] and therefore this paper was referenced to extract data pertaining to EE scores.

### 3.2 Study Characteristics

The characteristics of included studies are summarised in Table 1, with further detail provided in Supporting Information S1: Table S3. A total of 6729 participants (80.3% female) were included in this review; mean age was 45.8 (SD 9.4). The sample size ranged from 12 [61] to 1450 [83]. A full breakdown of ethnicity and gender can be found in Supporting Information S1: Tables S4 and S5. Twenty‐one studies were conducted in America [37, 39, 40, 41, 42, 43, 44, 45, 46, 47, 48, 50, 53, 56, 59, 61, 63, 67, 71, 80, 82], 10 studies were conducted in high‐income European countries (Netherlands, Finland, Italy, Portugal, Belgium, Spain, Switzerland) [51, 60, 65, 66, 68, 72, 73, 74, 76, 83], seven in middle‐income countries (Brazil, Turkey, Russia) [52, 57, 64, 69, 77, 78, 79], five studies were conducted in the UK [38, 54, 55, 58, 70] and four studies conducted in non‐European high‐income countries (Taiwan, Korea, Australia) [49, 62, 75, 81].

Interventions had a mean duration of 17 weeks (SD 19) with the shortest intervention being 1 day [51] and the longest intervention being 99 weeks [41]. Intervention types included 4 standard behavioural interventions (e.g., education, dietary and lifestyle recommendations, goal setting) [39, 57, 62, 83], 5 second‐wave CBT‐based interventions [49, 52, 74, 75, 81], 15 third‐wave CBT (e.g., ACT, Mindfulness‐Based Interventions, ME and Health at Every Size) [37, 38, 42, 43, 51, 54, 55, 59, 61, 67, 70, 76, 77, 78, 79] and 18 studies included a combination of approaches [40, 41, 44, 46, 47, 48, 50, 53, 56, 63, 64, 65, 68, 69, 71, 73, 80, 82]. The two most commonly utilised combinations of therapies were ACT and BWL [50, 56, 63, 65, 71] and Mindfulness and BWL [46, 48, 53, 80, 82]. The remaining five interventions targeted Appetitive Traits [45, 58], Intensive Counselling [60], Relaxation Techniques [66] and Food‐Based Activation [72].

Most interventions (*n* = 35, 74.5%) were group‐based and delivered in‐person [37, 39, 40, 41, 42, 43, 44, 47, 48, 50, 51, 52, 53, 54, 55, 56, 59, 60, 61, 62, 63, 65, 66, 68, 69, 71, 72, 73, 75, 76, 77, 79, 80, 81, 82], four studies (8.5%) were one to one and in‐person [45, 64, 74, 83], seven studies (14.9%) were one to one and remote [38, 46, 49, 57, 58, 67, 70] and one study (2.1%) [77] was a group‐based remotely delivered intervention.

### 3.3 Risk of Bias of Included Studies

The risk of bias in each study was assessed (see Supporting Information S1: Tables S3, S6 and S7). Of the 47 studies included, 28 (59.5%) were RCT [37, 38, 40, 41, 42, 43, 45, 46, 48, 50, 53, 57, 60, 62, 63, 64, 66, 69, 70, 72, 73, 74, 75, 78, 79, 80, 82, 83], 1 (2.1%) was a randomised crossover trial [49] and the remaining studies (*n* = 18, 38.4%) were quasi‐experimental in design [39, 44, 47, 51, 52, 54, 55, 56, 58, 59, 61, 65, 67, 68, 71, 76, 77, 81]. Of the randomised studies, 16 were low risk of bias [37, 38, 41, 45, 46, 50, 53, 63, 70, 72, 73, 74, 75, 79, 80, 82], 9 were some concern [40, 43, 48, 49, 57, 62, 64, 78, 83] and 4 were high risk of bias [42, 60, 66, 69]. Of the non‐randomised studies, 15 were assessed as having a moderate risk of bias [39, 44, 47, 51, 52, 56, 58, 59, 61, 68, 71, 76, 81] and three serious risk of bias. [54, 65, 77].

## 4 Meta‐Analysis

### 4.1 Pooled Effect of Interventions on Weight

**Figure 2:**
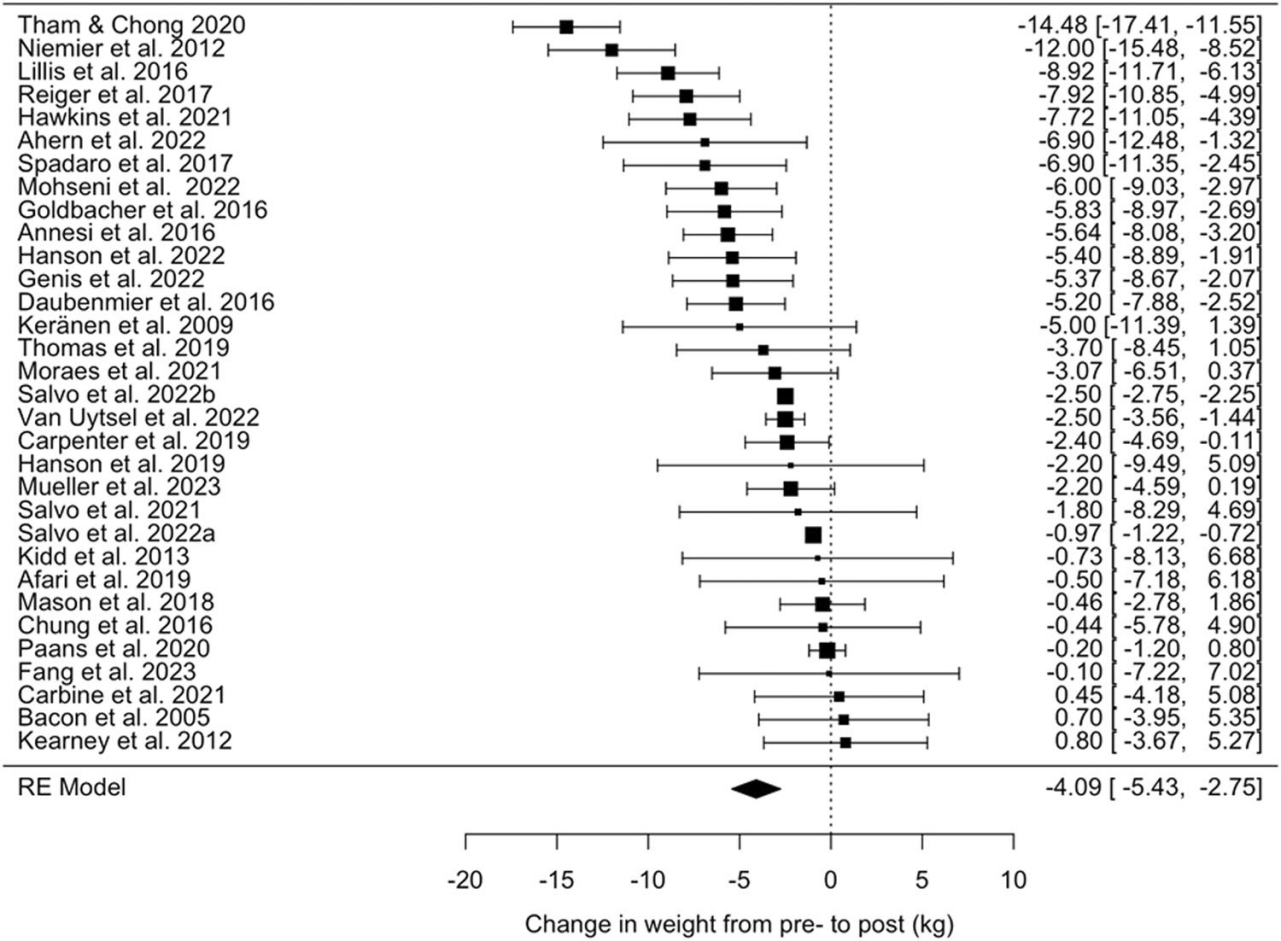
A forest plot demonstrating the change in weight from pre‐ to post‐intervention. The pooled effect of interventions on weight is −4.09 kg [95% CI: −5.43 to −2.76 kg], *p* < 0.001, *I*² = 96%, *τ*² = 11.06.

Thirty‐two studies contributed to the pooled effect estimate of interventions on weight, which was associated with a reduction in weight of −4.09 kg [95% CI: −5.43 to −2.76 kg], *p* < 0.001, *I* ^2^ = 96%, tau^2^ = 11.06 (see Figure 2). A Trim and Fill analysis imputed six effect sizes, which increased the pooled effect to −4.99 kg [95% CI: −6.29 to −3.65 kg]. Egger's test was not statistically significant (*Z* = 0.37, *p* = 0.714). Leave‐one‐out analyses did not lead to any substantial deviations from the overall pooled effect and all models remained significant. See Supporting Information S1: Figure S1 for a funnel plot illustrating the effect estimates for each study on weight, reflecting a low risk of publication bias.

### 4.2 Pooled Effect of Interventions on EE

**Figure 3:**
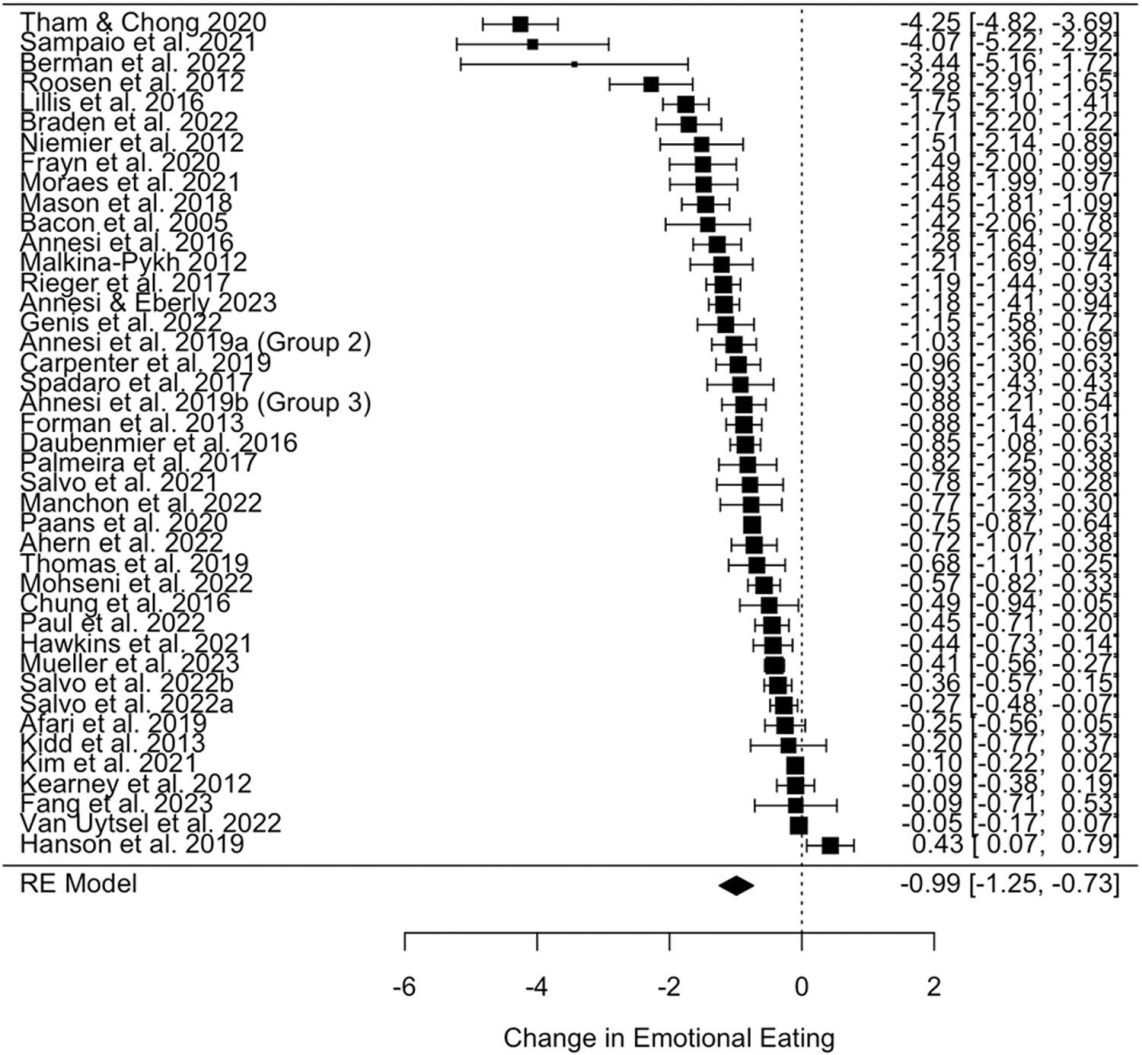
A forest plot demonstrating the change in standardised mean difference (SMD) score for emotional eating (EE) from pre‐ to post‐intervention. SMD = −0.99 [95% CI: −1.25 to −0.73], *p* < .001, *I*² = 97%, *τ*² = 0.68.

Forty‐two studies contributed to the pooled effect estimate on EE, collectively associated with a change in standardised mean difference (SMD) in EE score of −0.99 [95% CI: −1.25 to −0.73], *p* < 0.001, *I* ^2^ = 97%, tau^2^ = 0.68 (see Figure 3). Trim and Fill imputed 0 studies; however, Egger's test was statistically significant (*z* = 5.25, *p* < .001), suggesting a potential risk of bias. Analysis of boxplots demonstrated three clear outlying effect sizes with SMDs < −3.4 (see Supporting Information S1: Figure S2), and the removal of these changed the pooled estimated effect size of the SMD to −0.81 ([95% CI: −0.98 to −0.64], *p* < 0.001, *I* ^2^ = 93%, tau^2^ = 0.26). Outliers are removed in subsequent analyses. Therefore, the overall estimated effect indicates a large reduction in EE (SMD changes > 0.8 are deemed to be large effects), however, with considerable variability in effect sizes between studies.

### 4.3 BCTs

In total, 51 distinct BCTs were identified across the 46 studies. Five of these BCTs were used in less than three studies. The number of BCTs included in a single intervention ranged from 0 to 27. Only one study [49] did not provide sufficient detail to enable the identification of any BCTs. The most frequently utilised BCT was ‘instruction on how to perform the behaviour’ (*n* = 45), followed by ‘goal setting (behaviour)’ (*n* = 34), ‘problem solving’ (*n* = 34) and ‘reduce negative emotions’ (*n* = 34). A full list of BCTs and their estimated effect sizes and confidence intervals for weight and EE can be found in Supporting Information S1: Tables S8 and S9.

### 4.4 BCTs Associated With Effectiveness to Both Weight and EE

**Figure 4:**
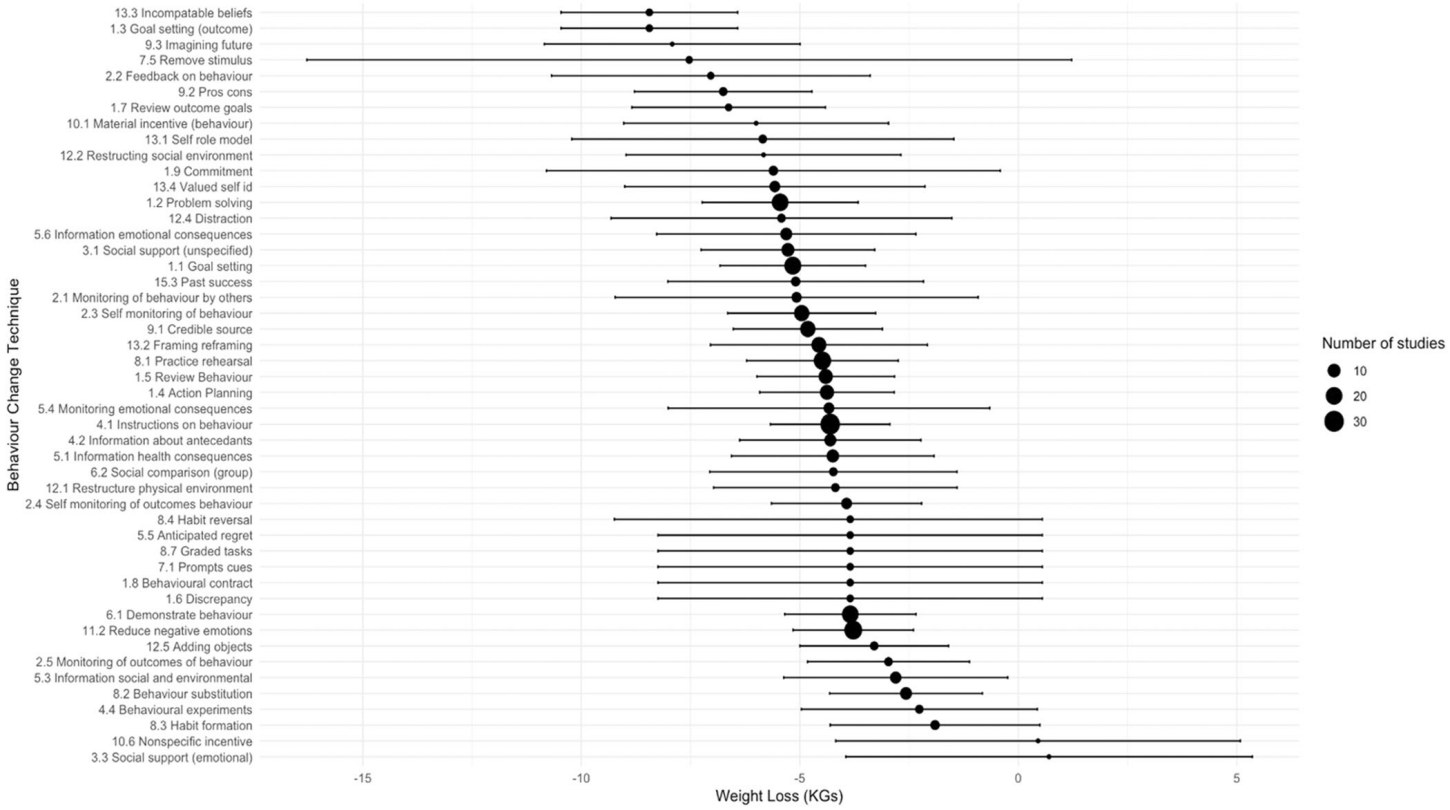
A forest plot showing the pooled effect size and 95% confidence intervals (CIs) for each behaviour change technique (BCT) on weight (kg), ranging from −8.44 kg [95% CI: −10.46 to −6.42] for BCT 13.3 (incompatible beliefs) to +0.7 kg [95% CI: −3.95 to 5.35] for BCT 3.3 (social support: emotional).

**Figure 5:**
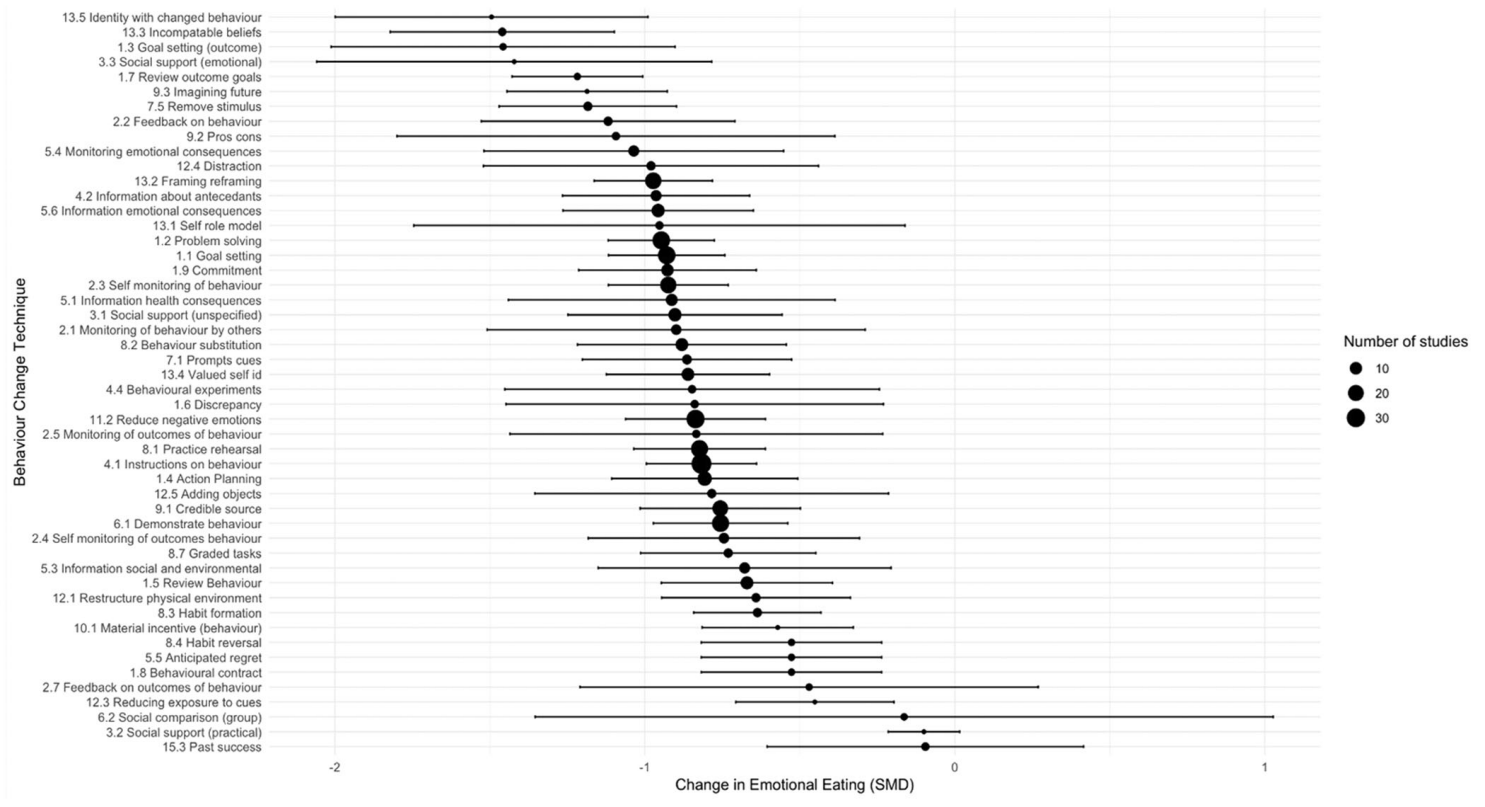
A forest plot showing the pooled effect size and 95% confidence intervals (CIs) for each behaviour change technique (BCT) on emotional eating (EE) ranging from −1.49 [95% CI: −2.00 to −0.99] for BCT 13.5 (identity associated with changed behaviour) to −0.01 [95% CI: −0.61 to 0.42] for BCT 15.3 (Focus on past successes).

Five BCTs (‘incompatible beliefs’, ‘goal setting outcome’, ‘review outcome goals’, ‘feedback on behaviour’ and ‘pros/cons’) were found in two or more studies and were associated with a statistically significant weight loss reduction ≥ 5 kg and reduction in SMD score ≥ 1. BCT ‘incompatible beliefs’ from cluster *identity* was associated with the largest reduction in weight (−8.44 kg [95% CI −10.46 to −6.42]) and SMD in EE score (−1.46 [95% CI −1.82 to −1.10] (see Figures 4, 5 and Supporting Information S1: Tables S8 and S9).

Another seven BCTs were also associated with statistically significant changes for weight and EE, but to a marginally lesser degree: ‘self as role model’, ‘commitment’, ‘problem solving’, ‘distraction’, ‘information about emotional consequences’, ‘social support unspecified’ and ‘goal setting (behaviour)’. These BCTs were associated with between 5 and 6 kg weight loss and a reduction in SMD score for EE of −0.93 to −0.98.

### 4.5 BCTs and Weight Only

**Figure 6:**
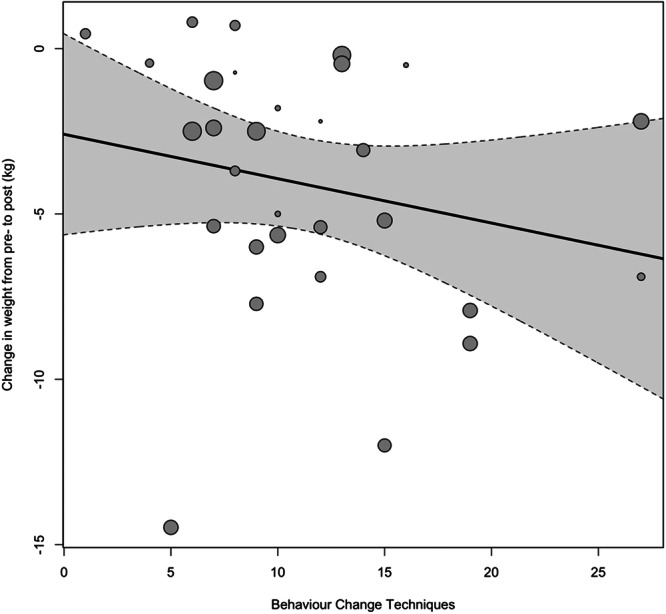
A regression plot of the number of identified BCTs against weight loss in the intervention group from pre‐ to post‐intervention, where the size of the individual points reflects the precision of the estimate (larger point = better precision).

There was no significant relationship between the number of BCTs and the study effect sizes for weight (*B* = −0.15 [95% CI: −0.37 to 0.10], *p* = 0.186: see Figure 6). For BCTs included in at least two or more studies, both ‘goal setting (outcome)’ and ‘incompatible beliefs’ were associated with the largest impact on weight reduction at −8.44 kg [95% CI: −10.46 to −6.42]. ‘Habit formation’ was associated with the smallest impact on weight reduction at −1.91 kg [95% CI −4.31 to 0.49] and was not statistically significant. Other BCTs associated with statistically significant reductions in weight > 5 kg include ‘valued self‐identity’, ‘focus on past successes’ and ‘monitoring of behaviour by others’ (see Figure 4 and Supporting Information S1: Table S8).

### 4.6 BCTs and EE Only

**Figure 7:**
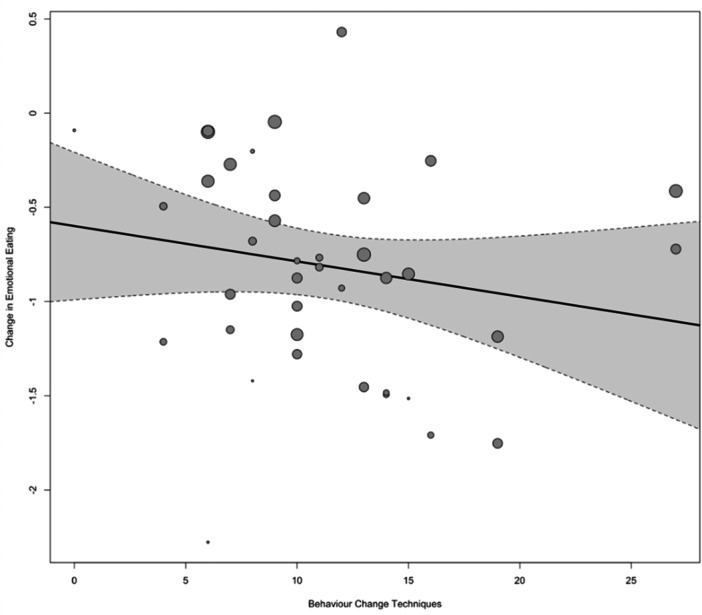
A regression plot of the number of identified BCTs against change in EE in the intervention group from pre‐ to post‐intervention, where the size of the individual points reflects the precision of the estimate (larger point = better precision).

There was no significant relationship between the number of BCTs and the study effect sizes for EE (B = −0.02 [95% CI: −0.05 to 0.01], *p* = 0.239: see Figure 7). The BCTs associated with the largest reduction in EE were: ‘incompatible beliefs’ (SMD = −1.46 [95% CI: −1.82 to −1.10]) and ‘goal setting (outcome)’ SMD = −1.46 [95% CI: −2.01 to −0.90]), whilst ‘focus on past successes’ was associated with the smallest impact and was not statistically significant (SMD −0.09 [95% CI −0.61 to 0.42]). The following BCTs, identified in two or more studies, were also among the higher performing BCTs for EE: ‘remove stimulus’, ‘monitoring of emotional consequences’, ‘framing/reframing’, ‘information about antecedents’, ‘self‐monitoring of behaviour’ and ‘information about health consequences’. These BCTs were associated with a reduction in SMD score of −0.91 to −1.18 (see Figure 5 and Supporting Information S1: Table S9).

### 4.7 Intervention Versus Control

To address concerns with the observational nature of the analysis, we compared post‐test scores between the intervention and control groups. Specifically, this involved excluding any BCTs that were common to both groups and assuming effect sizes for intervention effects were due to unique BCTs (those present in the intervention, but not the control group). The detailed results of this analysis are provided in the Supporting Information S1 (Section 7.0, Figures S3 and S4). This additional analysis corroborates findings regarding potential benefits of including self‐regulatory BCTs in EE interventions, whilst underscoring the added value of skills practice and rehearsal.

This analysis is not included in the main manuscript due to the need to exclude many BCTs used in both groups, limiting the ability to isolate the impact of individual BCTs. We provide recommendations for addressing these limitations in future studies to enhance the robustness of BCT and EE research (see Conclusion and Supporting Information S1: Table S11).

### 4.8 Other Outcomes of Interest

Due to a lack of sufficient data, we were unable to complete any analysis on the long‐term impact of interventions (at 12 months) and the impact of EE interventions on other measures of health (e.g. BP, cholesterol, HbA1C) as previously outlined in the protocol.

### 4.9 Sensitivity Analysis

Intervention delivery (in‐person vs. remote), format (group vs. individual vs. mixed), type of study (RCT or quasi‐experimental), length of intervention (in weeks) and quality of study did not impact changes in weight or EE score significantly (see Supporting Information).

### 4.10 GRADE Assessment

A Grading of Recommendations Assessment, Development and Evaluation (GRADE) assessment was undertaken and the degree of certainty for evidence pertaining to the overall impact of interventions on EE and weight was provided (see Supporting Information S1: Table S10). Overall certainty of evidence is low due to heterogeneity. Our recommendations are outlined in Supporting Information S1: Table S11.

## 5 Discussion

This review is an update and extension of a previous review [13] and to our knowledge, this is the first systematic review and meta‐analysis to investigate the impact of specific BCTs on EE and weight in adults living with overweight and obesity. Results were convergent with the original review, demonstrating a positive impact of psychological interventions with an EE component on weight and EE scores. Specifically, second‐ and third‐wave CBT approaches were found to be associated with positive effects. Regarding BCTs, results showed that the total number of BCTs within interventions was not associated with a greater effect, which has been observed elsewhere [90, 91]. Despite this, there were specific BCTs that were present in effective studies for both weight and EE outcomes, which suggest that the nature of BCTs is more important than quantity.

### 5.1 Identity and Future Self

BCTs belonging to the grouping ‘*Identity*’ appeared to be important to both outcomes, particularly ‘incompatible beliefs’. This involves creating discomfort by drawing attention to discrepancies between current or past behaviour and self‐image (cognitive dissonance) [36, 92] and has shown efficacy in another systematic review [93]. Furthermore, ‘incompatible beliefs’ has been found to improve attitudes towards positive behaviour change [94].

Other noteworthy ‘*Identity*’ focused BCTs, such as ‘self as role model’ and ‘valued self‐identity’, encourage individuals to contemplate self in a positive regard and promote reflection on values and actions that will benefit future self [95]. Psychological connectedness to future self‐identity has been found to be instrumental in behaviour change [96], especially regarding eating decisions [97]. Furthermore, a recent study of 344 adults [98] found that people experiencing body dissatisfaction and negative affect, both associated with EE [99, 100], will only engage in positive behaviour change when they have a strong connection to future self‐identity [98, 101]. Furthermore, being able to connect to the future self and a change in self‐narration has been shown to facilitate recovery from addiction [102]. BCT ‘information about health consequences’, which belongs to the grouping ‘*natural consequences’,* also encourages a focus on future health outcomes and positive consequences of action and was correlated with a significant change in EE.

Relatedly, ‘pros and cons’ involve assessing the consequences of action and were amongst BCTs associated with greater reductions in weight and EE. Despite it showing borderline significance and negative association with intervention effect in a systematic review of BCTs for healthy eating and physical activity [103], it has shown promise in a review of BCTs in alcohol and substance misuse [104], suggesting there are shared mechanisms between disordered eating and addictive behaviours [105]. This is supported by RIM and TTB theories of behaviour where individuals affected by trauma can improve psychological connectedness to their future self and develop reflective rather than automatic responses to triggers through trauma‐informed care [106]. ‘Framing/reframing’, also amongst the more significant BCTs for EE, involved observing thoughts and behaviours from a distance, allowing more deliberate and reflective choices. Collectively, this indicates that interventions emphasising identity, future self and consequences of action, may be important in addressing EE and other impulsive behaviours by encouraging more reflective approaches to behaviour change.

### 5.2 Self‐Regulation

In our review, self‐regulatory BCTs were found to be important to both EE and weight. BCT ‘goal setting (outcome)’, ‘goal setting (behaviour)’, ‘self‐monitoring of behaviour’, ‘feedback on behaviour’, ‘commitment’, ‘review outcome goals’ and ‘problem solving’ were all associated with significant but varying impacts on weight and EE. BCT ‘distraction’ was correlated with statistically significant changes to both weight and EE and has also shown efficacy in self‐management of binge eating [107]. Similar systematic reviews support evidence for self‐regulation BCTs [108, 109, 110]. The use of self‐regulatory skills is likely to increase an individual's feelings of efficacy about behaviour change, which in turn increases motivation and commitment for further change [111]. Furthermore, our review suggests a combination of both self‐driven regulation (e.g., problem solving, self‐monitoring) and external regulation (e.g., feedback on behaviour, monitoring of behaviour by others) are helpful in managing EE and weight.

### 5.3 Psychological Flexibility

BCT ‘removing aversive stimulus’ was associated with a significant reduction in EE score but not weight. In this review, this involved participants removing palatable foods from their homes or restricting access to palatable foods. This reflects conflicting evidence regarding whether dietary restraint is detrimental or beneficial to weight loss [112, 113]. For example, in certain individuals, restraint can exacerbate binge eating [114]. Adaptability appears to be important [113], with individuals who exhibit a more flexible approach to self‐regulation achieving greater success with weight loss and experiencing less disordered eating than those who adopt an ‘all or nothing’ approach [114]. Given that a lack of psychological flexibility is associated with EE [115, 116], interventions that encourage a flexible approach to self‐regulation may be advantageous. This focus on psychological flexibility [117] may explain why third‐wave CBT interventions such as ACT and mindfulness perform so well in EE interventions [13].

### 5.4 Self‐Compassion and Social Support

BCTs were associated with varying degrees of effectiveness to both outcomes, for example, ‘focusing on past successes’ appears to have produced the smallest reduction in EE compared to all other BCTs, however, is associated with relatively high weight loss. This may be explained by characteristics associated with EE, for example, holding oneself to high standards and being more sensitive to shame and self‐punishment [118], which may influence an individual's ability to recognise and celebrate past successes. Therefore, it is possible that individuals with EE may require support to develop self‐compassion skills before fully benefitting from this BCT. A recent systematic review and meta‐analysis found higher self‐compassion is associated with reduced disordered eating and self‐criticism [119]. Furthermore, ‘social support unspecified’ appears to foster self‐compassion [120] and has been found to reduce shame and stigma in addiction treatment [121]. Our review showed a strong association with reduction in EE and is amongst key strategies used in positive behaviour change for weight loss [122, 123], weight gain prevention [110] and self‐management of binge eating [107, 124]. Additionally, ‘social support (emotional)’, ‘information about antecedents’ and ‘monitoring emotional consequences’ were associated with significant reductions in EE, although ‘social support (emotional)’ was not significant for weight. Taken together, this indicates that an emphasis on understanding emotions (including antecedents of EE episodes) and adequate emotional support is needed in effective EE interventions.

### 5.5 Strengths and Limitations

This systematic review has several strengths given it is the first to explore BCTs and EE, and therefore presents novel findings. Generally, included studies provided a proficient level of detail to enable successful identification and extraction of BCTs. Furthermore, we have calculated the impact of each individual BCT on the outcomes of interest. The review has produced statistically significant results demonstrating which specific BCTs show promise to both weight management, EE and combined interventions, which can be applied to intervention development and further testing.

Several limitations should be considered. Whilst findings are positive, when a GRADE assessment was undertaken, it demonstrated an overall low level of certainty in evidence pertaining to weight changes and EE, due to high heterogeneity and risk of bias (see Supporting Information S1: 8.0 Grade Assessment). Furthermore, a pre–post analysis was most appropriate for examining individual BCTs due to the variability in control group type and how often similar BCTs were utilised in both the intervention and control groups. As such, we have made recommendations for further research (see Conclusion and Supporting Information S1: Table S11).

Second, it is important to consider the representativeness of the data; 80.3% of participants were female and 88.6% were either White or their ethnicity unknown, which reduces the ability to apply the findings to ethnically diverse populations who are most affected by obesity [125] but remain underrepresented in obesity research [126]. In addition, studies used self‐reported questionnaires which, it has been argued, may not be accurate measures of EE, as when assessed alongside direct measures of intake, they do not corroborate findings [127]. However, it is likely that feelings and behaviours aroused under experimental conditions are dissimilar to those aroused in real‐world settings. Therefore, self‐reported questionnaires are still warranted in eating behaviour research. Furthermore, the analysis considered only the contribution of individual BCTs but not the interaction between combinations of BCTs. Therefore, future work may consider a method such as qualitative comparative analysis (QCA) [128] that can ascertain not only the contribution of different conditions but also the combination of certain conditions. We were unable to investigate whether interventions impacted other health‐related outcomes and the long‐term effectiveness of interventions due to limited data on these outcomes. Recently, there has been a move towards a Behaviour Change Technique Ontology (BCTO) to improve the labels and definitions of BCTs, which should be applied to any future BCT research [129].

## 6 Conclusion and Future Directions

This is the first systematic review and meta‐analysis to examine how specific BCTs impact EE in adults living with overweight or obesity. Overall, interventions were associated with a significant reduction in weight loss and EE scores, but the substantial heterogeneity indicates that effectiveness varies widely and therefore there is low certainty of evidence. Further testing of the BCTs identified in this review, using high‐quality RCT design, is needed to strengthen confidence in the results. Future intervention studies addressing EE and weight should consider including the following BCTs as a minimum: ‘incompatible beliefs’, ‘goal setting (outcome)’, ‘pros/cons’, ‘review outcome goal’ and ‘feedback on behaviour’. Interventions may also benefit from the inclusion of ‘commitment’, ‘goal setting behaviour’, ‘information about emotional consequences’, ‘distraction’, ‘self as role model’, 'problem solving’ and ‘social support unspecified’. Interventions that address EE only should consider the following additional BCTs: ‘social support (emotional), ‘information about health consequences’, ‘remove aversive stimulus’ and ‘monitoring emotional consequences’.

Our recommendations are that clinicians consider how best to screen patients for the presence of EE and tailor advice accordingly. Policymakers should consider funding the development of further psychological support in weight management interventions that address EE. EE interventions are likely to benefit from a TTB emphasis, with a focus on self‐regulation skills, psychological flexibility, self‐identity and values whilst developing self‐compassion skills. Future research should focus on an agreed definition of EE and consistent screening tools and consider both positive and negative EE. Future EE intervention studies must strengthen their reporting of BCTs and specify the components of their intervention according to the TIDieR template, which would allow for more consistent terminology and analysis.

## Author Contributions

D.P., J.M., G.T.‐T., and L.E. led the overall conceptualisation and design. D.P., P.D., and A.G. completed title and paper screening with support from J. S. A. J. led the design and completion of data analysis. D.P. completed TIDieR extraction with support from K.S. BCT extraction and coding was completed by D.P. and C.K. All authors contriuted to interpretation of findings. D.P. prepared the manuscript with input from all authors.

## Conflicts of Interest

The authors declare no conflicts of interest.

### 1 Peer Review

The peer review history for this article is available at https://www.webofscience.com/api/gateway/wos/peer-review/10.1111/jhn.13410.

## Supporting information

Supporting information.

## Data Availability

The data that support the findings of this study are openly available in the OSF data repository at https://osf.io/6bfdj/.
