# Supplementary material for: Emotional Eating Interventions for Adults Living With Overweight and Obesity: A Systematic Review and Meta‐Analysis of Behaviour Change Techniques
Source: J Hum Nutr Diet. 2025 Jan 6;38(1):e13410. doi: 10.1111/jhn.13410 (PMC11704659; doi:10.1111/jhn.13410)
Supplement: Supplementary file 1 — Supporting information. [file JHN-38-0-s001.docx]

**Emotional Eating interventions for adults living with overweight and obesity: A systematic review and meta-analysis of behaviour change techniques.**

**Supplementary Materials**

# Contents

[Contents 2](#_Toc179815629)

[1.0 PRISMA Checklist 3](#_Toc179815630)

[2.0 Search strategy 11](#_Toc179815631)

[2.1 Reasons for Exclusion 11](#_Toc179815632)

[3.0 Characteristics of Included Studies 15](#_Toc179815633)

[3.1 Ethnicity 31](#_Toc179815634)

[3.2 Gender 33](#_Toc179815635)

[4.0 Risk of Bias 34](#_Toc179815636)

[4.1 RoB2 34](#_Toc179815637)

[4.2 ROBINS-I 35](#_Toc179815638)

[5.0 Behaviour Change Techniques 36](#_Toc179815639)

[6.0 Sensitivity analysis**:** 38](#_Toc179815640)

[6.1 Weight change. 38](#_Toc179815641)

[6.2 Emotional Eating 39](#_Toc179815642)

[6.3 Publication bias 40](#_Toc179815643)

[7.0 BCT analysis Intervention vs. Control 41](#_Toc179815644)

[8.0 GRADE Assessment 44](#_Toc179815645)

[9.0 Recommendations 46](#_Toc179815646)

# 1.0 PRISMA Checklist

**Supplementary Table S1: Systematic Review PRISMA checklist.**

| **Section and Topic** | **Item #** | **Checklist item** | **Location where item is reported (section)** |
| --- | --- | --- | --- |
| **TITLE** | | |  |
| Title | 1 | Identify the report as a systematic review. | Title |
| **ABSTRACT** | | |  |
| Abstract | 2 | See the PRISMA 2020 for Abstracts checklist. | Abstract |
| **INTRODUCTION** | | |  |
| Rationale | 3 | Describe the rationale for the review in the context of existing knowledge. | 1.0 Introduction |
| Objectives | 4 | Provide an explicit statement of the objective(s) or question(s) the review addresses. | 1.0 Introduction |
| **METHODS** | | |  |
| Eligibility criteria | 5 | Specify the inclusion and exclusion criteria for the review and how studies were grouped for the syntheses. | 2.2 Eligibility criteria  2.2.5 Study design  2.3 Search strategy |
| Information sources | 6 | Specify all databases, registers, websites, organisations, reference lists and other sources searched or consulted to identify studies. Specify the date when each source was last searched or consulted. | 2.3 Search strategy |
| Search strategy | 7 | Present the full search strategies for all databases, registers and websites, including any filters and limits used. | 2.2 Eligibility criteria  2.3 search strategy  2.3.1 Screening process |
| Selection process | 8 | Specify the methods used to decide whether a study met the inclusion criteria of the review, including how many reviewers screened each record and each report retrieved, whether they worked independently, and if applicable, details of automation tools used in the process. | 2.3.1 Screening process  Figure 1: PRISMA flow chart Supplementary materials, Table S2 Reasons for exclusion |
| Data collection process | 9 | Specify the methods used to collect data from reports, including how many reviewers collected data from each report, whether they worked independently, any processes for obtaining or confirming data from study investigators, and if applicable, details of automation tools used in the process. | 2.4 Data Extraction |
| Data items | 10a | List and define all outcomes for which data were sought. Specify whether all results that were compatible with each outcome domain in each study were sought (e.g. for all measures, time points, analyses), and if not, the methods used to decide which results to collect. | 2.2 Eligibility criteria  2.3 search strategy  2.4 Data Extraction |
|  | 10b | List and define all other variables for which data were sought (e.g. participant and intervention characteristics, funding sources). Describe any assumptions made about any missing or unclear information. | 2.2 Eligibility criteria  2.3 search strategy  2.4 data extraction  2.6 Data synthesis |
| Study risk of bias assessment | 11 | Specify the methods used to assess risk of bias in the included studies, including details of the tool(s) used, how many reviewers assessed each study and whether they worked independently, and if applicable, details of automation tools used in the process. | 2.5 Risk of Bias |
| Effect measures | 12 | Specify for each outcome the effect measure(s) (e.g. risk ratio, mean difference) used in the synthesis or presentation of results. | Section 4.0 Meta-analysis  Figure 2 and Figure 3  Supplementary materials: Table S3 Summary of study characteristics, Table S8 and S9- Impact of each BCT on outcomes weight and EE |
| Synthesis methods | 13a | Describe the processes used to decide which studies were eligible for each synthesis (e.g. tabulating the study intervention characteristics and comparing against the planned groups for each synthesis (item #5)). | 2.2 Eligibility criteria  2.3 search strategy  2.3.1 Screening process  Supplementary Table S2 Reasons for exclusion |
|  | 13b | Describe any methods required to prepare the data for presentation or synthesis, such as handling of missing summary statistics, or data conversions. | 2.6 Data Analysis |
|  | 13c | Describe any methods used to tabulate or visually display results of individual studies and syntheses. | Figure 2: Forest Plot for change in weight by each study  Figure 3: Forest plot demonstrating the change in SMD score for EE by each study  Figure 4: Forest Plot showing the pooled effect size and 95% CIs for each BCT on weight loss  Figure 5: Forest Plot showing the pooled effect size and 95% CIs for each BCT on EE  Table 1: Summary of Included Studies  Table S3:Summary of study characteristics |
|  | 13d | Describe any methods used to synthesize results and provide a rationale for the choice(s). If meta-analysis was performed, describe the model(s), method(s) to identify the presence and extent of statistical heterogeneity, and software package(s) used. | 2.6 Data Analysis  4.0 Meta-analysis  4.3 Behaviour Change Techniques |
|  | 13e | Describe any methods used to explore possible causes of heterogeneity among study results (e.g. subgroup analysis, meta-regression). | 4.9 Sensitivity Analysis  Supplementary materials 6.0 |
|  | 13f | Describe any sensitivity analyses conducted to assess robustness of the synthesized results. | 4.9 Sensitivity analysis  Supplementary Materials 6.0 |
| Reporting bias assessment | 14 | Describe any methods used to assess risk of bias due to missing results in a synthesis (arising from reporting biases). | 2.6 Data analysis  4.1 and 4.2 Pooled effect of interventions on weight and EE  3.3 Risk of bias of included studies.  Supplementary materials: Table S6 RoB2 and Table S7 ROBINS-I  Supplementary Materials: Figure S1, Figure S2 Funnel Plots |
| Certainty assessment | 15 | Describe any methods used to assess certainty (or confidence) in the body of evidence for an outcome. | 4.1 and 4.2 Pooled effect of interventions on weight and EE  4.3 Behaviour Change Techniques  4.9 Sensitivity analysis  Supplementary Materials: Figure S1, S2 publication bias  6.0 Sensitivity Analysis  Table S10 GRADE Assessment |
| **RESULTS** | | |  |
| Study selection | 16a | Describe the results of the search and selection process, from the number of records identified in the search to the number of studies included in the review, ideally using a flow diagram. | Figure 1: PRISMA flow chart |
|  | 16b | Cite studies that might appear to meet the inclusion criteria, but which were excluded, and explain why they were excluded. | Figure 1: PRISMA flow chart  Supplementary materials, Table S2 Reasons for exclusion |
| Study characteristics | 17 | Cite each included study and present its characteristics. | Table 1: Summary of Included Studies  Table S3: Summary of study characteristics |
| Risk of bias in studies | 18 | Present assessments of risk of bias for each included study. | Supplementary Materials Table S6, and Table S7 |
| Results of individual studies | 19 | For all outcomes, present, for each study: (a) summary statistics for each group (where appropriate) and (b) an effect estimate and its precision (e.g. confidence/credible interval), ideally using structured tables or plots. | Table 1, summary of included studies Table S3 Summary of study characteristics  Figure 2: Forest plot demonstrating the change in weight (kg) from pre- to post-intervention, ordered by size  Figure 3: Forest plot demonstrating the change in SMD score for EE from pre- to post-intervention  Figure 4: Forest plot demonstrating the pooled effect size and 95% CIs for each BCT on weight loss.  Figure 5: Forest plot demonstrating the pooled effect size and 95% CIs for each BCT on EE  Supplementary Materials Table S8 and S9  Figure 6: A regression plot of the number of identified BCTs against weight loss in the intervention group from pre- to post-intervention  Figure 7: A regression plot of the number of identified BCTs against change in EE in the intervention group from pre- to post-intervention  Supplementary Materials 7.0 Intervention vs. Control, Figure S3, Figure S4 |
| Results of syntheses | 20a | For each synthesis, briefly summarise the characteristics and risk of bias among contributing studies. | Table 1: Summary of Included Studies,  3.2 Study Characteristics  Supplementary materials: Table S3: Characteristics of Included studies  Table S10 GRADE assessment |
|  | 20b | Present results of all statistical syntheses conducted. If meta-analysis was done, present for each the summary estimate and its precision (e.g. confidence/credible interval) and measures of statistical heterogeneity. If comparing groups, describe the direction of the effect. | 4.1 Pooled effect of interventions on weight  4.2 Pooled effect of interventions on EE  4.7 Intervention Vs. Control  Figure 2: Forest Plot demonstrating change in weight (Kg) by intervention, ordered by size  Figure 3: Forest plot demonstrating the change in SMD score for EE by intervention study, ordered by size  Figure 4: Forest plot demonstrating the pooled effect size and 95% CIs for each BCT on weight loss  Figure 5: Forest plot demonstrating the pooled effect size and 95% CIs for each BCT on EE  Figure 6: A regression plot of the number of identified BCTs against weight loss in the intervention group from pre- to post-intervention  Figure 7: A regression plot of the number of identified BCTs against change in EE in the intervention group from pre- to post-intervention  Supplementary materials: 7.0 Intervention vs. Control, Figure S3 and Figure S4 |
|  | 20c | Present results of all investigations of possible causes of heterogeneity among study results. | 4.1 Pooled effect of interventions on weight  4.2 Pooled effect of interventions on EE  4.9 Sensitivity analysis  Supplementary material 6.0 |
|  | 20d | Present results of all sensitivity analyses conducted to assess the robustness of the synthesized results. | 4.1 Pooled effect of interventions on weight  4.2 Pooled effect of interventions on EE  4.9 Sensitivity analysis  Supplementary material 6.0 |
| Reporting biases | 21 | Present assessments of risk of bias due to missing results (arising from reporting biases) for each synthesis assessed. | Supplementary materials Table S6 and Table S7 |
| Certainty of evidence | 22 | Present assessments of certainty (or confidence) in the body of evidence for each outcome assessed. | 4.1 Pooled effect of interventions on weight  4.2 Pooled effect of interventions on EE  Figure 2: Forest Plot for change in weight by intervention study, ordered by size  Figure 3: Forest plot for change in EE by intervention study, ordered by size |
| **DISCUSSION** | | |  |
| Discussion | 23a | Provide a general interpretation of the results in the context of other evidence. | 6. Discussion |
|  | 23b | Discuss any limitations of the evidence included in the review. | 6.1 Strengths and Limitations, Supplementary Materials Table S10 GRADE assessment |
|  | 23c | Discuss any limitations of the review processes used. | 6.1 Strengths and Limitations, Supplementary Materials Table S10 GRADE assessment |
|  | 23d | Discuss implications of the results for practice, policy, and future research. | 7. Conclusion and future directions  Supplementary Materials, Table S11 |
| **OTHER INFORMATION** | | |  |
| Registration and protocol | 24a | Provide registration information for the review, including register name and registration number, or state that the review was not registered. | 2. Methods |
|  | 24b | Indicate where the review protocol can be accessed, or state that a protocol was not prepared. | 2.1 Protocol and Registration |
|  | 24c | Describe and explain any amendments to information provided at registration or in the protocol. | 4.8 Other Outcomes of interest |
| Support | 25 | Describe sources of financial or non-financial support for the review, and the role of the funders or sponsors in the review. | Acknowledgments |
| Competing interests | 26 | Declare any competing interests of review authors. | Acknowledgments |
| Availability of data, code and other materials | 27 | Report which of the following are publicly available and where they can be found: template data collection forms; data extracted from included studies; data used for all analyses; analytic code; any other materials used in the review. | Reference list  Supplementary Materials |

# 2.0 Search strategy

Ebsco (CINAHL, MEDLINE, PsychInfo) <**Limiters** - Publication Date: 20220101-20240431>

S1. (‘’Obes*’’ OR ‘’overweight’’ or ‘’weight’’ OR ‘’bmi’’ OR ‘’Body mass index’’ OR ‘’waist circumference’’)

S2. (‘’adult’’ OR ‘’over 18’’)

S3. (‘’Mindful*’’ OR ‘’mindful eat*’’ OR ‘’Emotional Eating’’ OR ‘’cognitive behavio*’’ OR ‘’Behavio* change’’ OR ‘’binge eat*’’ OR ‘’comfort eat*’’ OR ‘’self-help’’ OR ‘’food addiction’’ OR ‘’Acceptance and Commitment Therapy’’ OR ‘’ACT’’)

S4. (‘’Intervention*’’ OR ‘’treatment*’’)

S5. (‘’Weight loss’’ OR ‘’weight reduction’’ OR ‘’lose weight’’ OR ‘’eating control’’)

S6. S1 AND S2 AND S3 AND S4 AND S5 AND S6

Embase <**Limiters** - Publication Date: 20220101-20240431>

1. (‘’Obes*’’ OR ‘’overweight’’ or ‘’weight’’ OR ‘’bmi’’ OR ‘’Body mass index’’ OR ‘’waist circumference’’)
2. (‘’adult’’ OR ‘’over 18’’)
3. (‘’Mindful*’’ OR ‘’mindful eat*’’ OR ‘’Emotional Eating’’ OR ‘’cognitive behavio*’’ OR ‘’Behavio* change’’ OR ‘’binge eat*’’ OR ‘’comfort eat*’’ OR ‘’self-help’’ OR ‘’food addiction’’ OR ‘’Acceptance and Commitment Therapy’’ OR ‘’ACT’’)
4. (‘’Intervention*’’ OR ‘’treatment*’’)
5. (‘’Weight loss’’ OR ‘’weight reduction’’ OR ‘’lose weight’’ OR ‘’eating control’’)
6. 1 AND 2 AND 3 AND 4 AND 5 AND

## 2.1 Reasons for Exclusion

**Table S2: Reasons for Exclusion**

| Number | Reference | Summary Comment for exclusion |
| --- | --- | --- |
| 1. | Saslow LR, Missel AL, O’Brien A, Kim S, Hecht FM, Moskowitz JT, Bayandorian H, Pietrucha M, Raymond K, Richards B, Liestenfeltz B. Psychological Support Strategies for Adults With Type 2 Diabetes in a Very Low–Carbohydrate Web-Based Program: Randomized Controlled Trial. JMIR diabetes. 2023 May 11;8:e44295. | No psychological component targeting EE |
| 2. | Radin RM, Epel ES, Mason AE, Vaccaro J, Fromer E, Guan J, Prather AA. Impact of digital meditation on work stress and health outcomes among adults with overweight: A randomized controlled trial. PloS one. 2023 Mar 1;18(3):e0280808. | This study focused on binge eating, not EE. |
| 3. | Monterubio GE. *Testing of a Novel Combined Eating-Disorder and Weight-Loss Online Guided-Self Help Intervention for Young Adults with a Binge-Type Eating Disorder and Overweight or Obesity* (Doctoral dissertation, Washington University in St. Louis). | Intervention was for treatment of BED. |
| 4. | Hopkins CM. *Reduction of Internalized Weight Bias via Mindful Self-Compassion: Theoretical Framework and Results from a Randomized Controlled Trial* (Doctoral dissertation, Duke University). | Not published in a peer reviewed journal |
| 5. | Dauber A, Redondo R, Meschino KJ, Braden A. Dietary changes in an acceptance-based weight loss pilot intervention study. Eating Behaviors. 2023 Jan 1;48:101707. | Secondary research |
| 6. | Kanda A, Sugimura Y, Ohishi H, Tatebayashi S, Sawada K, Wai KM, Nishiguchi K, Tanabu A, Jung S, Murashita K, Nakaji S. Body Compression Corrective Garment and Eating Behavioural Change for Weight Reduction: The Mutsu City Randomised Controlled Trial. InHealthcare 2023 Mar 24 (Vol. 11, No. 7, p. 942). MDPI. | No psychological component targeting EE |
| 7. | Cifuentes L, Ghusn W, Feris F, Campos A, Sacoto D, De la Rosa A, McRae A, Rieck T, Mansfield S, Ewoldt J, Friend J. Phenotype tailored lifestyle intervention on weight loss and cardiometabolic risk factors in adults with obesity: a single-centre, non-randomised, proof-of-concept study. EClinicalMedicine. 2023 Apr 1;58. | Some information missing to allow for inclusion, no response from author. |
| 8. | Bricker JB, Mull KE, Sullivan BM, Forman EM, Lillis J, McTiernan A, Santiago-Torres M. Telehealth acceptance and commitment therapy for weight loss: Protocol of the WeLNES full scale randomized controlled trial. Contemporary clinical trials. 2023 Mar 1;126:107091. | Protocol only, no published results |
| 9. | Butryn ML, Crane NT, Lufburrow E, Hagerman CJ, Forman EM, Zhang F. The Role of Physical Activity in Long-term Weight Loss: 36-month Results From a Randomized Controlled Trial. Annals of Behavioral Medicine. 2023 Feb 1;57(2):146-54. | Some information missing to allow for inclusion, no response from author |
| 10. | Bauman V. *Acceptability of an Acceptance-Based Behavioral Weight-Loss Intervention for Adults in Rural Areas* (Doctoral dissertation, University of Florida). | Doctoral dissertation, not published in peer reviewed journal. |
| 11. | Barchfeld DC, Vagi RK, Lüdtke K, Schieffer E, Güler F, Einecke G, Jäger B, de Zwaan M, Nöhre M. Cognitive-behavioral and dietary weight loss intervention in adult kidney transplant recipients with overweight and obesity: Results of a pilot RCT study (Adi-KTx). Frontiers in Psychiatry. 2023 Apr 11;14:1071705. | No EE specific component. |
| 12. | Salvo V, Sanudo A, Kristeller J, Schveitzer MC, Martins P, Favarato ML, Demarzo M. Mindful eating for overweight and obese women in Brazil: An exploratory mixed-methods pilot study. Nutrition and Health. 2022 Dec;28(4):591-601. | Already included in original review |
| 13. | Hooker AR, Sagui-Henson SJ, Daubenmier J, Moran PJ, Hartogensis W, Acree M, Kristeller J, Epel ES, Mason AE, Hecht FM. Effects of a mindfulness-based weight loss intervention on long-term psychological well-being among adults with obesity: Secondary analyses from the Supporting Health by Integrating Nutrition and Exercise (SHINE) Trial. Mindfulness. 2022 Sep;13(9):2227-42. | Secondary analysis of data already included in the systematic review |
| 14. | Halali F, Lapveteläinen A, Aittola K, Männikkö R, Tilles-Tirkkonen T, Järvelä-Reijonen E, Absetz P, Kolehmainen M, Schwab U, Lindström J, Lakka TA. Associations between weight loss history and factors related to type 2 diabetes risk in the Stop Diabetes study. International Journal of Obesity. 2022 May;46(5):935-42. | Some information missing to allow for inclusion, no response from author |
| 15. | Guerrero-Hreins E, Stammers L, Wong L, Brown RM, Sumithran P. A comparison of emotional triggers for eating in men and women with obesity. Nutrients. 2022 Oct 6;14(19):4144. | Secondary analysis |
| 16. | Tobin SY, DeSalvo K, Smith JK, Beachman A, Chamberlain E, Cornier MA, Halliday TM. A Feasibility Trial To Determine The Effect Of A Mindfulness Intervention On Weight-loss Maintenance: 1955. Medicine & Science in Sports & Exercise. 2022 Sep 1;54(9S):581. | Congress meeting |
| 17. | Nunes CL, Carraça EV, Jesus F, Finlayson G, Francisco R, Silva MN, Santos I, Bosy‐Westphal A, Martins P, Minderico C, Sardinha LB. Changes in food reward and intuitive eating after weight loss and maintenance in former athletes with overweight or obesity. Obesity. 2022 May;30(5):1004-14. | Intervention did not target EE or measure EE specifically |
| 18. | Fitzsimmons‐Craft EE, Chan WW, Smith AC, Firebaugh ML, Fowler LA, Topooco N, DePietro B, Wilfley DE, Taylor CB, Jacobson NC. Effectiveness of a chatbot for eating disorders prevention: a randomized clinical trial. International Journal of Eating Disorders. 2022 Mar;55(3):343-53. | No psychological component targeting EE |
| 19. | Cox JS, Searle A, Hamilton-Shield J, Hinton EC. The development of an ACT-based intervention for self-determination in weight management. Appetite. 2022 Dec 1;179:106226. | Qualitative analysis of a weight management intervention, without an EE focus |
| 20 | Cardi V, Meregalli V, Di Rosa E, Derrigo R, Faustini C, Keeler JL, Favaro A, Treasure J, Lawrence N. A community-based feasibility randomized controlled study to test food-specific inhibitory control training in people with disinhibited eating during COVID-19 in Italy. Eating and Weight Disorders-Studies on Anorexia, Bulimia and Obesity. 2022 Oct;27(7):2745-57. | >90% of the study population had a diagnosis of an ED |
| 21. | Carbine KA, Muir AM, Allen WD, LeCheminant JD, Baldwin SA, Jensen CD, Kirwan CB, Larson MJ. Does inhibitory control training reduce weight and caloric intake in adults with overweight and obesity? A pre-registered, randomized controlled event-related potential (ERP) study. Behaviour Research and Therapy. 2021 Jan 1;136:103784. | Already included in original systematic review by Smith et al. (2023) |
| 22. | Burrell CJ. *Impact of an obesity intervention in a southern rural community* (Doctoral dissertation, Concordia University Chicago). | Dissertation and no EE focus |
| 23. | Burns R, Firman E, Huang HC. Assessing service provision and outcomes at the Canberra Obesity Management Service: A retrospective chart review. Obesity. 2022 Nov;30(11):2146-55. | No psychological component targeting EE |
| 24. | Boutelle KN, Eichen DM, Peterson CB, Strong DR, Kang-Sim DJ, Rock CL, Marcus BH. Effect of a novel intervention targeting appetitive traits on body mass index among adults with overweight or obesity: A randomized clinical trial. JAMA Network Open. 2022 May 2;5(5):e2212354-. | No psychological component targeting EE |
| 25. | Bates S, Norman P, Breeze P, Brennan A, Ahern AL. Mechanisms of action in a behavioral weight-management program: latent growth curve analysis. Annals of Behavioral Medicine. 2022 Jan 1;56(1):64-77. | No psychological component targeting EE |
| 26. | Abdul Basir SM, Abdul Manaf Z, Mazri FH, Mat Ludin AF, Shahar S, Abdul Manaf MR. Description of a Hybrid Mindfulness-Integrated Multidisciplinary Workplace Weight Management Intervention Module ‘Mind-SLIMSHAPE’Using the TIDieR Checklist. Nutrients. 2022 Jul 29;14(15):3140. | Quantitative data related to intervention not published yet, author responded to contact and said would be in touch when paper is published. |

# 3.0 Characteristics of Included Studies

**Table S3 Summary of study characteristics, results and risk of bias**

| **Author, Date, Intervention Name and Country** | **Study Characteristics** | **Intervention to Address EE** | **Outcomes Reported, EE tool and results** | **Risk of bias, Tool, score** |
| --- | --- | --- | --- | --- |
| **Afari et al. (2019)**  **MOVE + ACT**  **USA** | Design: RCT  Sample size: n = 88 (85 completers)  Mean age: 57.3 years (SD: 9.9).  Gender: 76.1% male,  Ethnicity: 70.5% Caucasian, 17% African American, and 12% Hispanic | Interventions:  Acceptance and Commitment Therapy (ACT)  Behavioural Weight Loss (BWL)  Both length of interventions: 8 weeks in person, 90 days by telephone  Settings: In person and by telephone  Delivered by: (ACT) - A full-time staff psychologist; 2 psychology postdoctoral fellows; a psychology master’s student; and ACT therapists. (BWL) - A full-time staff psychologist; a psychology postdoctoral fellow and a psychology master’s student | Weight and EE, DEBQ  Change in SMD score for EE and 95% CI= -0.25 [-0.56, 0.05]  Change in Weight (kg) and 95% CI = -0.5 [-7.18, 6.18] | RoB2, Low risk |
| **Ahern et al. (2022)** | Design: RCT  Sample size: n = 61  Mean age: 48  Ethnicity: 95% Caucasian, 84% female | Interventions:  Web-based, Guided Self-Help based on ACT principles.  Compared to Standard advice  Length of intervention: 12 weeks, individual  Delivered by: Non-specialist SWiM coaches | Weight and EE, DEBQ  Change in SMD score for EE and 95% CI= -0.72 [-1.07, -0.38]  Change in Weight (kg) and 95% CI = -6.90 [-12.48, -1.32] | RoB2, Low risk |
| **Annesi & Eberly (2023)**  **USA** | Design: Single Group  Sample size: 121  Mean age: Not provided, participant age groupings were established for analyses as emerging adult (18–24.9 years, n=35), young adult (25–43.9 years, n=33), and middle-aged adult (44–59.9 years, n=53)  Gender: 100% Female  Ethnicity: 72% White, 25% Black, 3% other | Intervention:  Community-based obesity treatment centered around self-regulatory skills to control eating  Intervention length: 6 months, in person | EE only, EES  Change in SMD score for EE and 95% CI= -1.18 [-1.41, -0.94] | ROBINS-I, Moderate risk |
| **Annesi, et al. (2016)**  **LEARN**  **USA** | Design: Individually randomized group treatment trial Sample size: n = 103  Mean age: 47.8 years (SD: 7.9)  Gender: 100% female  Ethnicity: 84% White, 12% African American, and 4% other | Interventions:  CBT + BWL (personal contact group vs. self-help).  Length of the intervention: 6 months, in person  Delivered by: A trained and nationally certified wellness professional | Weight and EE, EES  Change in SMD score for EE and 95% CI= -1.28 [-1.64, -0.92]  Change in Weight (kg) and 95% CI = -5.64 [-8.08, -3.20] | RoB2, Some concern |
| **Annesi (2019)**  **USA** | Design: Individually randomized group treatment trial Sample size: n = 152  Mean age: 48.6 years (SD:7.0)  Gender: 100% female  Ethnicity: 80% White, 15% Black, 5% other | Interventions:  Group 1: Behavioural Weight Loss (BWL)  Group 2: Behavioural Weight Loss (BWL)  + Cognitive Behavioural Treatment (CBT)  Group 3: Behavioural Weight Loss (BWL)  + Cognitive Behavioural Treatment (CBT).  Length of interventions:  Group 1: 28 weeks, phone calls  Group 2: 58 weeks, in person  Group 3: 99 weeks, phone calls and in person  Delivered by: All intervention were delivered by the authors | EE only, EES  Change in SMD score for EE and 95% CI=  Group 2: -1.03 [-1.36, -0.69]  Group 3: -0.88 [-1.21, -0.54] | RoB2, Low risk |
| **Bacon et al. (2005)**  **Health at Every Size**  **USA** | Design: Individually randomized group treatment trial  Sample size: n = 35 completers at post-intervention.  Mean age: HAES: 40.4 (SD:4.4); Diet: 41.4 years (SD:3.0)  Gender: 100% female  Ethnicity: Not reported | Interventions:  Diet Group (BWL)  Health at Every Size (HAES) Group (Acceptance-based)  Length of intervention: 6 months, group  Delivered by: The Diet Group program was taught by an experienced Registered Dietitian. The Health at Every Size Group was facilitated by a counsellor who had conducted educational and psychotherapeutic workshops | Weight and EE, EI  Change in SMD score for EE and 95% CI= -1.42 [-2.06, -0.78]  Change in Weight (kg) and 95% CI = 0.7 [-3.95, 5.35] | RoB2, High risk |
| **Berman et al. 2022**  **USA** | Design: RCT  Sample size: n = 19, 15 completers at post-intervention.  Mean age: 51 years  Gender: 100% Female  Ethnicity: Caucasian 100% | Interventions:  Health at Every Size (HAES) Group and ACT combination  Compared to Weight Watchers  Lenth of intervention: 11 weeks  Delivered by: Health at Every Size Expert | BMI and EE, EDDS  Change in SMD score for EE and 95% CI= -3.44 [-5.16, -1.72] | RoB 2, some concern |
| **Braden et al. 2022**  **USA** | Design: Open Trial (Pilot)  Sample size: n = 39, with 35 completers  Mean age: 49.21 (SD:10.91)  Gender: 100% Female  Ethnicity 92.3% Caucasian | Intervention:  DBT skills and behavioral weight loss techniques  Length of intervention: 16 weeks, first session was individual, followed by group sessions  Delivered by: Psychologists | BMI and EE, BES  Change in SMD score for EE and 95% CI= -1.71 [-2.20, -1.22] | ROBINS-I, Moderate |
| **Carbine et al. (2021)**  **USA** | Design: RCT  Sample size: n = 100  Mean age: 28.05 years (SD:7.56)  Gender: 53% Female  Ethnicity: 81% Caucasian, 14% Hispanic, 5% other | Interventions:  Food Specific ICT (i.e., inhibiting responses to high-calorie foods) (Other therapy)  Generic ICT (i.e., inhibiting responses to everyday items) (Other therapy)  Length of intervention: 4 weeks, in person  Delivered by: The authors | Weight and EE, DEBQ  Change in Weight (kg) and 95%= 0.45 [-4.18, 5.08]  Unable to use EE data | RoB2, Low risk |
| **Carpenter et al. (2019)**  **Mind Your Weight**  **USA** | Design: Individually randomized group treatment trial, pilot study  Sample size: n = 75  Mean age: 47.3 years (SD:10.0)  Gender: 92% Female  Ethnicity: 65.3% White, 26.7% Black, 6.7% Hispanic, 1.3% Asian | Interventions:  Mindfulness weight loss program (Mind Your Weight) (Mindfulness)  Behavioural weight loss program (Weight TalkTM) (BWL)  Length of intervention: 6 months, telephone-based counselling  Both delivered by: Health Coaches and Registered Dietitians | Weight and EE, MEQ  Change in SMD score for EE and 95% CI: -0.96 [-1.30, -0.63]  Change in Weight (kg) and 95%= -2.40 [-4.69, -0.11] | RoB2, Low risk |
| **Chung et al. (2016)**  **USA** | Design: Single group design, longitudinal study  Sample size: n = 22  Mean age: 50.14 years (SD:9.0)  Gender: 100% Female  Ethnicity: 100% African American | Intervention: Mindful Eating  Length of intervention: 24 weeks, group and in person and telephone calls  Delivered by: Registered Dietitian | Weight and EE, MEQ  Change in Weight (kg) and 95%= -0.44 [-5.78, 4.90]  Change in SMD score for EE and 95% CI: -0.49 [-0.94, -0.05] | ROBINS-I, Moderate |
| **Daubenmier et al. (2016)**  **SHINE**  **USA** | Design: RCT  Sample size: n = 194  Mean age: 47.5 years (SD:12.7)  Gender: 80% Female  Ethnicity: 59.3% European, 12.9% African, 9.8% Asian/Pacific Islander, 11.9% Latina/Latino, 1% Native American, 5.1% other | Intervention:  Mindfulness-based weight loss intervention  Behavioural Weight Loss as active control group  Length of intervention: Both interventions included 16 sessions lasting 2 to 2.5 h (12 weekly, 3 biweekly, and 1 monthly) and one all-day session (6.5 and 5 h in the Mindfulness and control interventions, respectively) over 5.5 months.  Delivered by: Registered Dietitians, with the Mindfulness being delivered by Mindfulness Meditation Instructors. | Weight only reported, EE data reported in and extracted from Mason et al.2016^(45)^  Change in Weight (kg) and 95%= -5.2 [-7.88, -2.52]  Change in SMD score for EE and 95% CI: -0.85 [-1.08, -0.63] | RoB2, Some concern |
| **Fang et al. (2023)**  **Taiwan** | Design: Randomized cross over pilot  Sample size: n = 20  Mean age: 42  Gender: 75% Female, 25% Male  Ethnicity: Not reported | Intervention:  Telehealth assisted intervention on weight reduction, mood status, and eating behavior change under a smartphone application (app) with 3D food picture recognition and incorporated with cognitive behavioral training programs.  Length of intervention: 4 weeks, phone based  Delivered by: App-based | Weight and EE, MEBS  Unable to use data for weight  Change in SMD score for EE and 95% CI: -0.09 [-0.71, 0.53] | RoB2, Some concern |
| **Forman et al. (2013)**  **Mind your Health**  **USA** | Design: RCT  Sample size: n = 128 (99 completers post-intervention)  Mean age: 45.69 years (SD:12.81)  Gender: Not reported  Ethnicity: 62.3% Caucasian, 24.6% African American, 1.6% Asian, 3.8% Hispanic | Interventions:  Acceptance-based Behavioural Treatment (Acceptance-based)  Standard Behavioural Treatment (Behavioural therapy)  Length of intervention: 40 weeks, group and in person  Delivered by: Psychologist | EE only, EES  Unable to use EE data | RoB2, some concern |
| **Frayn et al. (2020)**  **Switzerland** | Design: Single group design  Sample size: n = 32  Mean age: 46.71 years (SD:13.43)  Gender: 87.5% Female, 12.5% Male  Ethnicity: 78.1% Caucasian, 3.1% Middle Eastern, 3.1% Black, 3.1% Hispanic, 12.5% other | Intervention:  Acceptance and Commitment Therapy (Acceptance-based)  Length of intervention: 1 day, group and in person  Delivered by: A PhD candidate in clinical psychology who had training in ACT. | EE only, DEBQ  Change in SMD score for EE and 95% CI: -1.49 [-2.00, -0.99] | ROBINS-I, Moderate |
| Geniş **et al. (2022)**  **Turkey** | Design: Single Group Pilot  Sample size: n = 40, 35 completers  Mean age: 41.71 years (SD: 4.46)  Gender: 91.4% of completers Female, 8.6% of completers Male  Ethnicity: Not reported | Intervention:  CBT  Length of intervention: 8 weeks  Delivered by: MDT | Weight and EE, DEBQ  Change in Weight (kg) and 95%= -5.37 [-8.67, -2.07]  Change in SMD score for EE and 95% CI: -1.15 [-1.58, -0.72] | ROBINS-I, Moderate |
| **Goldbacher et al. (2016)**  **USA** | Design: Individually randomized group treatment trial  Sample size: n = 79  Mean age: 45.6 years (SD:10.5)  Gender: 95% Female, 5% Male  Ethnicity: 80% African American, 11% White, 4% Hispanic, 5% other | Interventions:  Behavioural Weight Loss Treatment (BWL)  Enhanced Behavioural Treatment (Behavioural Therapy)  Length of intervention: 20 weeks, group and in person  Both delivered by: Masters- and doctoral-level clinicians. | Weight and EE, EES  Change in Weight (kg) and 95%= -5.83 [-8.97, -2.69]  Unable to use EE data (post SD missing) | RoB2, Low risk |
| **Hanson et al. (2018)**  **UK** | Design: Single group design  Sample size: n = 53 (33 completers)  Mean age: 44.4 years (SD:11.0)  Gender: 78.8% Female  Ethnicity: Not reported  Mean weight: 126.3 kg (SD:36.1) | Intervention:  Mindfulness  Compared to control group  Length of intervention: 8 weeks, group and in person  Delivered by: A team of specialist dietitians, psychologists, and physicians. | Weight and EE, WPIEQ  Change in Weight (kg) and 95%= -2.20 [-9.49, 5.09]  Change in EE score and 95% CI= 0.43 [0.07- 0.79] | ROBINS-I, Serious risk |
| **Hanson et al. (2022)**  **CALMPOD**  **UK** | Design: Retrospective Cohort analysis  Sample size: n = 289  Mean age: 46.9 years (SD: 11.3)  Gender: 74.4% Female  Ethnicity: Not reported | Intervention:  Mindfulness  Compared to control group  Length of intervention: 8 weeks, group and in person  Delivered by: MDT | Weight only  Change in Weight (kg) and 95%= -5.40 [-8.89, -1.91] | ROBINS-I, Moderate |
| **Hawkins et al. (2021)**  **POWER-UP**  **USA** | Design: Single group design  Sample size: n = 48  Mean age: 43.58 years (SD:1.50)  Gender: 85% Female  Ethnicity: Not reported | Intervention:  Acceptance-based Behavioural Therapy (Acceptance-based)  Length of intervention: 23 weeks, group and in person  Delivered by: A nurse trained in BWL and a clinical psychologist. | Weight and EE, EES  Change in Weight (kg) and 95%: -7.72 [-11.05,-4.39]  Change in SMD score for EE and 95% CI: -0.44 [-0.73, -0.14] | ROBINS-I, Moderate |
| **Hepdurgun et al. (2020)**  **Turkey** | Design: Individually randomized group treatment trial  Sample size: n = 51 in the intervention group  Mean age: 40.1 years (SD:9.96)  Gender: 80.4% Female, 19.6% Male  Ethnicity: Not reported | Intervention:  Internet-based Behavioural Therapy  Compared to routine care (healthy eating and physical activity information) by email  Length of intervention: 8 weeks, online and in person  Delivered by: Online | Weight only  Change in Weight (kg) and 95%:  Unable to use weight as data incomplete | RoB2, some concern |
| **Hunot-Alexander et al. (2021)**  **UK** | Design: Single group design with qualitative evaluation  Sample size: n = 37 (weight data reported for 32)  Mean age: 48.3 years (SD:10.9)  Gender: 93.8% Female, 6.3% Male  Ethnicity: 90.6% White, 9.4% non-White | Intervention:  Appetitive Trait Tailored Intervention (Other therapy)  Length of intervention: 8 weeks, in person  Delivered by: A PhD student | BMI only  Unable to use data as incomplete | ROBINS-I, Moderate |
| **Kearney et al. (2012)**  **USA** | Design: Single group design  Sample size: n = 48 (38 completers post-intervention)  Mean age: 49 years (SD:10.7)  Gender: 87.5% Male  Ethnicity: 85.4% White, 4.2% Black, 6.3% Hispanic, 4.2% Asian/Pacific-Islander/Native American | Intervention:  Mindfulness-based stress reduction (Mindfulness).  Length of intervention: 2 months, group and in person  Delivered by: Instructors who met professional guidelines for teaching MBSR | Weight and EE, TFEQ  Change in Weight (kg) and 95%: 0.8 [-3.67, 5.27]  Change in SMD score for EE and 95% CI: -0.09 [-0.38, 0.19] | ROBINS-I, Moderate |
| **Keränen et al. (2009)**  **LITE**  **Finland** | Design: Individually randomized group treatment trial  Sample size: n = 20 completers in the intervention group  Mean age: 52 years (SD: 7.0)  Gender: 25% Male  Ethnicity: Not reported | Intervention:  Intensive Counselling (with components of EE)  Compared to Short-term Counselling (no components of EE)  Length of intervention: 20 weeks, group and in person  Delivered by: A Clinical Nutritionist | Weight only  Change in Weight (kg) and 95%: -5.00 [-11.39, 1.39] | RoB2, High risk |
| **Kidd et al. (2013)**  **USA** | Design: Single group design  Sample size: n = 12  Mean age: 51.8 years (SD: 9.1)p  Gender: 100% Female  Ethnicity: 58.3% African American, 41.7% White | Intervention:  Mindful Eating  Length of intervention: 8 weeks, in person  Delivered by: The Research Team | Weight and EE, MEQ  Change in Weight (kg) and 95%: -0.73 [-8.13, 6.68]  Change in SMD score for EE and 95% CI: -0.20 [-0.77, 0.37] | ROBINS-I, Moderate |
| **Kim et al. (2021)**  **Healthy Life Plan**  **Korea** | Design: RCT  Sample size: n = 583 (369 completers post-intervention)  Mean age: 53.68 years (SD:10.12) for the IG and 53.94 years (SD: 10.18) for the MG.  Gender: 61.6% Female, 38.4% Male  Ethnicity: Not reported | Intervention:  Intensive intervention group (IG). Received a multi-component intervention to reduce abdominal obesity by mainly focusing on dietary attitude and dietary behaviour change and a minimal information intervention (Other therapy)  Minimal information intervention group Received a brief explanation of health status and a simple recommendation for a lifestyle change (Other therapy)  Length of intervention: 6 months, group and in person  Delivered by: Trained Clinical Nutritionists | EE only, DEBQ  Change in SMD score for EE and 95% CI: -0.10 [-0.22, 0.02] | RoB2, some concern |
| **Lillis et al. (2016)**  **USA** | Design: RCT  Sample size: n = 162  Mean age: 50.2 years (SD: 10.9)  Gender: 85% Female, 15% Male  Ethnicity: 5% Black/African American, 6% Hispanic, 1% Asian, 88% Caucasian | Intervention:  Standard Behavioural Treatment (Behavioural Therapy)  Acceptance-based Behavioural Intervention (ABBI Acceptance-based)  Length of intervention: 24 months, group and in person  Delivered by: A Ph.D. psychologist; a Ph.D. exercise physiologist; and a master’s level nutritionist. | Weight and EE, EI  Change in Weight (kg) and 95%: -8.92 [-11.84, -6.00]  Change in SMD score for EE and 95% CI: -1.75 [-2.10, -1.41] | RoB2, Low risk |
| **Malkina-Pykh (2012)**  **Russia** | Design: RCT  Sample size: n = 104 (58 completers at post-intervention)  Mean age: 37.6 years (SD:6.7)  Gender: 69% Female, 31% Male  Ethnicity: Not reported | Intervention:  Cognitive Behavioural Therapy (CBT) +/−  Rhythmic Movement Therapy (RMT). This was added for half of participants who showed no improvement with CBT after 6 months (n = 30). The remaining 28 participants who did not respond to CBT after 6 months continued with CBT.  Length of intervention: 24 bi-weekly sessions (48 weeks), in person  Delivered by: Psychologists trained in CBT | EE only, DEBQ  Change in SMD score for EE and 95% CI: -1.21 [-1.69,-0.74] | RoB2, some concern |
| **Manchón et al. (2022)**  **Spain** | Design: Single Group intervention study  Sample size: n = 23, with 9 completers  Mean age: 44.11 years (SD: 5.82)  Gender: 100% Female  Ethnicity: Not reported | Intervention:  Acceptance and commitment therapy (ACT) and Behavioural weight loss  Length of intervention: 10 weeks | EE only, PNEES  Change in SMD score for EE and 95% CI: -1.77 [-1.23,-0.30] | ROBINS-I, Serious |
| **Manzoni et al. (2009)**  **Italy** | Design: RCT  Sample size: n = 40 in the two intervention groups  Mean age: Not reported  Gender: 100% Female  Ethnicity: Not reported | Intervention:  Relaxation training—traditional (imagination condition)  Relaxation training (virtual reality condition)  Compared to standard hospital-based care  Length of intervention: 5 weeks, in person during an inpatient stay  "Delivered by:  Two licensed clinical psychologists and one licensed psychotherapist under the supervision of a senior psychotherapist" | Weight and EE, EOQ  Unable to use data as incomplete for this analysis | RoB2, High risk |
| **Mason et al. (2018)**  **USA** | Design: Single group design  Sample size: n = 104 (61 completers at post-intervention)  Mean age: 46.07 years (SD:14.64)  Gender: Not reported  Ethnicity: 68.3% White, 4.8% Black, 10.6% Hispanic/Latino, 9.6% Asian/Pacific Islander, 0.0% Native American, 1.0% declined to answer | Interventions:  Mindfulness  Length of intervention: 28 days, via a mobile app  Delivered by: Video lectures and guidance on Mindfulness practice. | Weight and EE, FCQ-T-R  Change in Weight (kg) and 95%: -0.46 [-2.78,1.86]  Change in SMD score for EE and 95% CI: -1.45 [-1.81, -1.09] | ROBINS-I, Moderate |
| **Mohseni et al. (2022)**  **Netherlands** | Design: Single Group Pilot  Sample size: n = 96  Mean age: 42 years (SD: 13.0)  Gender: 76% Female  Ethnicity: 84,4% Caucasian | Intervention:  Multidisciplinary combined lifestyle intervention (CLI) and CBT  Length of intervention: 1.5 years | Weight and EE, DEBQ  Change in Weight (kg) and 95%: -6.00 [-9.03,-2.97]  Change in SMD score for EE and 95% CI: -0.57 [-0.82, -0.33] | ROBINS-I, Moderate |
| **Moraes et al. (2021)**  **Brazil** | Design: RCT  Sample size: n = 64 in the two intervention groups of interest  Mean age: EH: 35.98 years (SD:6.76); IT + CBT: 36.18 years (SD:2.75)  Gender: EH: 81.8% Female, 18.2% Male; IT +CBT: 77.4% Female, 22.6% Male  Ethnicity: Not reported | Interventions:  Education and Health Group (EH) (BWL)  Interdisciplinary Therapy plus Cognitive Behavioural Therapy (IT + CBT)  Compared to a physical activity program (no elements of EE, control)  Length of intervention: 30 weeks, group (EH), group and individual (IT + CBT)  Delivered by: Health professionals | Weight and EE, DEBQ  Change in Weight (kg) and 95%: -3.07 [-6.51, 0.37]  Change in SMD score for EE and 95% CI: -1.48 [-1.99, -0.97] | RoB2, High risk |
| **Mueller et al. 2022**  **UK** | Design: RCT  Sample size: n = 192 in the intervention group  Mean age: 50.7 years (SD: 14.3)  Gender: 78.1% Female  Ethnicity: 93.8% white | Interventions:  Web-based, Guided Self-Help based on ACT principles  Compared to Standard advice  Length of intervention: 12 weeks, individual  Delivered by: Wellness Professional | Weight and EE, TFEQ  Change in Weight (kg) and 95%: -2.20 [-4.59, 0.19]  Change in SMD score for EE and 95% CI:-0.41 [-0.56, -0.27] | RoB2, Low risk |
| **Niemeier et al. (2012)**  **USA** | Design: Single group design pilot  Sample size: n = 21 (18 completers at post-intervention)  Mean age: 52.2 years (SD:7.6)  Gender: 90.5% Female, 9.5% Male  Ethnicity: 90% non-Hispanic, 4.8% Hispanic, 4.8% other | Intervention:  Acceptance-Based Behavioural Intervention (ABBI BWL + Acceptance-based)  Length of intervention: 24 weeks, group  Delivered by: A PhD- level Clinical Psychologist; a Clinical Psychology Intern; and a masters-level nurse with expertise in behavioural weight loss. | Weight and EE, EI  Change in Weight (kg) and 95%: -12.00 [-15.48, -8.52]  Change in SMD score for EE and 95% CI: -1.41 [-2.01, -0.81] | ROBINS-I, Moderate |
| **Paans et al. (2020)**  **MooDFOOD**  **Netherland** | Design: RCT  Sample size: n = 372 for intervention group  Mean age: 47.8 years (SD:12.6)  Gender: 78.2% Female  Ethnicity: Not reported | Intervention:  Food-related Behavioural Activation Therapy (Behavioural approach)  Groups:  Multi-nutrient supplement + FBA  Placebo supplement + FBA  Multi-nutrient supplement  Placebo supplement  Length of intervention: 1 year, 15 individual sessions and 6 group sessions  Delivered by: Psychologists familiar with behavioural activation. | Weight and EE, TFEQ  Change in Weight (kg) and 95%: : -0.20 [-1.20, 0.80]  Change in SMD score for EE and 95% CI: -0.75 [-0.87, -0.64] | RoB2, Low risk |
| **Palmeira et al. (2017)**  **Kg-free**  **Portugal** | Design: RCT  Sample size: n = 27 for intervention group  Mean age: 41.97 years (SD: 8.79).  Gender: 100% women  Ethnicity: Not reported | Intervention:  Acceptance and Commitment Therapy (Acceptance-based)  Compared to treatment as usual  Length of intervention: 12 weeks, group and in person  Delivered by: A Clinical Psychologist with previous training in contextual-behavioural therapies and a clinical psychology master’s student. | EE only, TFEQ  Change in SMD score for EE and 95% CI: -0.82 [-1.25, -0.38] | RoB2, Low risk |
| **Paul et al (2022)**  **Netherlands** | Design: RCT  Sample size: n = 130  Mean age: 41.4 years (SD: 9.8)  Gender: 74.6% female  Ethnicity: Not reported | Intervention:  CBT group was compared to a treatment-as-usual (TAU) control group  Length of intervention: 10 weeks  Delivered by: CBT Therapist | BMI and EE, DEBQ  Change in SMD score for EE and 95% CI: -0.45[ -0.71, -0.2) | RoB2, Low risk |
| **Rieger et al. (2017)**  **Australia** | Design: RCT  Sample size: n = 201 (118 completers post-intervention)  Mean age: 47.01 years (SD:11.52)  Gender: 73.6% Female | Intervention:  Cognitive Behaviour Therapy (CBT):  Weight loss alone (CBT)  Weight loss with the addition of a Support Person (CBT).  Length of intervention: 12 months, group and in person  Delivered by: Five therapists with postgraduate degrees in clinical psychology. | Weight and EE, BES  Change in Weight (kg) and 95%: -7.92 [-10.85, -4.99]  Change in SMD score for EE and 95% CI: -1.19 [-1.44, -0.93] | RoB2, Low risk |
| **Roosen et al. (2012)**  **Netherlands** | Design: Pilot, single group design  Sample size: n = 35  Mean age: 39.2 years (SD:11.02)  Gender: 86% Female, 15% Male  Ethnicity: Not reported | Intervention:  Dialectical Behaviour Therapy (DBT)  Length of intervention: 20 weeks, group and in person  Delivered by: Two trained co- therapists | EE only, DEBQ  Change in SMD score for EE and 95% CI: -2.28 [-2.91, -1.65] | ROBINS-I, Moderate |
| **Salvo et al. (2021)**  **MB-EAT**  **Brazil** | Design: Pilot, single group design, with mixed-methods evaluation  Sample size: n = 20  Mean age: 48.15 years (SD:8.57)  Gender: 100% Female  Ethnicity: Not reported | Intervention:  Mindfulness-based Eating Awareness Training (Mindfulness)  Length of intervention: 13 weeks, group  Delivered by: Research Team | Weight and EE, EAT  Change in Weight (kg) and 95%: -1.80 [-8.29, 4.69]  Change in SMD score for EE and 95% CI: -0.78 [-1.29, -0.28] | ROBINS-I, Serious |
| **Salvo et al. (2022)**  **Brazil** | Design: RCT  Sample size: n = 284  Mean age: 40.4 years (SD : 10.7)  Gender: 100% Female  Ethnicity: Not reported | Interventions:  Mindful eating programme compared to a mindfulness programme and a no-treatment control group.  Length of intervention: 10 weeks, group and in person  Delivered by: researcher who has specific meditation training and practice | Weight and EE, DEBQ  Change in Weight (kg) and 95%, Group A: -0.98 [-1.22, -0.72]  Change in SMD score for EE and 95% CI, Group A: -0.27 [-0.48, -0.07]  Group B: -0.36 [-0.57, -0.15] | RoB2, some concern |
| **Sampaio et al. (2021)**  **Brazil** | Design: RCT  Sample size: n = 27 for intervention group  Mean age: 49 years (SD:11.0)  Gender: 100% Female  Ethnicity: 7.4% White, 40.7% Black, 51.9% mixed | Intervention:  Meditation practice. It presumes that health is related to balance and integration of the physiological, emotional, cognitive, behavioural, and spiritual aspects of human functioning (Mindfulness)  Compared to a control group  Length of intervention: 7 months, group  Delivered by: A Nutritionist, a family doctor, a Gynaecologist, and an Endocrinologist. | EE only, DEBQ  Change in SMD score for EE and 95% CI: -4.07 [-5.22, -2.92] | RoB2, Low risk |
| **Spadaro et al. (2017)**  **USA** | Design: RCT  Sample size: n = 46  Mean age: 45.2 years (SD:8.2)  Gender: 87% Female, 13% Male  Ethnicity: 78.3% Caucasian, 21.7% African American | Intervention:  Behavioural Weight Loss Programs (BWL)  Behavioural Weight Loss Program + Mindfulness Meditation (BWL + Mindfulness)  Length of intervention: 6 months, group  Delivered by: A doctoral student in exercise physiology | Weight and EE, EI  Change in Weight (kg) and 95%: -6.9 [-11.35, -2.45]  Change in SMD score for EE and 95% CI: -0.93 [-1.43, -0.43] | RoB2, Low risk |
| **Tham and Chong (2020)**  **Medical & Mind Weight Loss Redefine CBT Programme**  **Australia** | Design: Single group design  Sample size: n = 120  Mean age: Not reported  Gender: 57.5% Female, 42.5% Male | Intervention:  Cognitive Behavioural Therapy (CBT)  Length of intervention: 26 weeks, online  Delivered by: The research team through online resources. | Weight and EE, EEQ  Change in Weight (kg) and 95%: -14.48 [-17.41, -11.55]  Change in SMD score for EE and 95% CI: -4.25 [-4.82, -3.69] | ROBINS-I, Moderate |
| **Thomas et al. (2019)**  **POWER and MORE POWER**  **USA** | Design: RCT  Sample size: n = 51  Mean age: 57.92 years (SD: 10.04)  Gender: 100% Female  Ethnicity: 96% White, 2% Black/African American, 2% Hispanic/Latino | Intervention:  Exercise and nutrition counselling POWER (BWL)  Exercise and nutrition counselling + MOREPOWER (Mindfulness)  Length of intervention: 10 weeks, group and in person  Delivered by: A Registered Dietitian and a master’s- level licensed clinical social worker. | Weight and EE, DEBQ  Change in Weight (kg) and 95%: -3.70 [-8.45, 1.05]  Change in SMD score for EE and 95% CI: -0.68 [-1.11, -0.25] | RoB2, Low risk |
| **Van Uytsel (2022)**  **InterACT**  **Belgium** | Design: RCT  Sample size: n =1075, completion rate of 76%  Mean age: 31.2 (SD: 4.0)  Gender: 100% Female | Intervention:  Post partum Lifestyle intervention, e-health and face to face focusing on nutrition, exercise and mental wellbeing and using motivational interviewing and behaviour change techniques  Control group received no intervention  Delivered by: Lifestyle Coach | Weight and EE, TFEQ-R18  Change in Weight (kg) and 95%: -2.50 [-3.56, -1.44]  Change in SMD score for EE and 95% CI: -0.05 [-0.17, 0.07] | RoB2, some concern |

## 3.1 Ethnicity

**Table S4 Ethnicity**

| Author | Country | Sample size | White/ Caucasian | Black/ African american | Hispanic/ latino | Asian | Native American | More than one race | Other | Prefer not to say | Not reported |
| --- | --- | --- | --- | --- | --- | --- | --- | --- | --- | --- | --- |
| Ahern 2022 | UK | 61 | 58 |  |  |  |  |  |  |  | 3 |
| Afari et al. 2019 | USA | 88 | 62 | 17 | 12 |  |  |  |  |  |  |
| Annesi & Eberly 2023 | USA | 121 | 87 | 30 |  |  |  |  | 4 |  |  |
| Annesi et al. 2016 | USA | 103 | 87 | 12 |  |  |  |  | 4 |  |  |
| Annesi et al. 2019 | USA | 152 | 122 | 23 |  |  |  |  | 7 |  |  |
| Geniş et al. 2020 | Turkey | 35 |  |  |  |  |  |  |  |  | 35 |
| Bacon et al. 2005 | USA | 78 |  |  |  |  |  |  |  |  | 78 |
| Berman et al. 2022 | USA | 19 | 19 |  |  |  |  |  |  |  |  |
| Braden et al. 2022 | USA | 39 | 36 |  |  |  |  |  |  |  | 3 |
| Carbine et al. 2021 | USA | 100 | 81 |  | 14 |  |  |  | 5 |  |  |
| Carpenter et al. 2019 | USA | 75 | 49 | 20 | 5 | 1 |  |  |  |  |  |
| Chung et al. 2016 | USA | 22 |  | 22 |  |  |  |  |  |  |  |
| Daubenmier et al. 2016 | USA | 194 |  | 25 | 23 | 19 | 2 |  | 125 |  |  |
| Fang et al. 2023 | Taiwan | 20 |  |  |  |  |  |  |  |  | 20 |
| Forman et al. 2013 | USA | 128 | 81 | 31 | 5 | 2 |  |  |  |  | 10 |
| Frayn et al. 2020 | Switzerland | 32 | 25 | 1 | 1 |  |  |  | 5 |  |  |
| Goldbacher et al. 2016 | USA | 79 | 9 | 63 | 3 |  |  | 1 | 3 |  |  |
| Hanson et al. 2022 | UK | 289 |  |  |  |  |  |  |  |  | 289 |
| Hanson et al. 2019 | UK | 53 |  |  |  |  |  |  |  |  | 53 |
| Hawkins et al. 2021 | USA | 48 |  |  |  |  |  |  |  |  | 48 |
| Hepdurgan et al. 2020 | Turkey | 51 |  |  |  |  |  |  |  |  | 51 |
| Hunot-Alexander et al. 2021 | UK | 53 | 48 |  |  |  |  |  | 5 |  |  |
| Kearney et al. 2012 | USA | 48 | 41 | 2 | 3 | 2 |  |  |  |  |  |
| Keränen et al. 2009 | Finland | 82 |  |  |  |  |  |  |  |  | 82 |
| Kidd et al. 2013 | USA | 12 | 5 | 7 |  |  |  |  |  |  |  |
| Kim et al. 2021 | Korea | 583 |  |  |  |  |  |  |  |  | 583 |
| Lillis et al. 2016 | USA | 162 | 142 | 8 | 10 | 2 |  |  |  |  |  |
| Malkinah-Pykh 2012 | Russia | 104 |  |  |  |  |  |  |  |  | 104 |
| Manchón et al. 2022 | Spain | 23 |  |  |  |  |  |  |  |  | 23 |
| Manzoni et al. 2009 | Italy | 40 |  |  |  |  |  |  |  |  | 40 |
| Mason et al. 2018 | USA | 104 | 71 | 5 | 11 | 10 |  |  | 6 |  | 1 |
| Mohseni et al. 2022 | Netherlands | 96 | 81 |  |  |  |  |  |  |  | 15 |
| Moraes at al. 2021 | Brazil | 64 |  |  |  |  |  |  |  |  | 64 |
| Mueller et al. 2022 |  | 388 | 364 |  |  |  |  |  | 21 | 3 |  |
| Niemier et al. 2012 | USA | 21 |  |  | 1 |  |  |  | 20 |  |  |
| Paans et al. 2020 | Netherlands | 743 |  |  |  |  |  |  |  |  | 743 |
| Palmeira et al. 2017 | Portugal | 27 |  |  |  |  |  |  |  |  | 27 |
| Paul et al. 2022 | Netherlands | 130 |  |  |  |  |  |  |  |  | 130 |
| Reiger et al. 2017 | Australia | 201 |  |  |  |  |  |  |  |  | 201 |
| Roosen et al. 2019 | Netherlands | 35 |  |  |  |  |  |  |  |  | 35 |
| Salvo et al. 2021 | Brazil | 20 |  |  |  |  |  |  |  |  | 20 |
| Sampaio et al. 2021 | Brazil | 55 | 5 | 23 |  |  |  | 27 |  |  |  |
| Spadaro et al. 2017 | USA | 46 | 36 | 10 |  |  |  |  |  |  |  |
| Tham & Chong 2020 | Australia | 120 |  |  |  |  |  |  |  |  | 120 |
| Thomas et al. 20 | USA | 51 | 49 | 1 | 1 |  |  |  |  |  |  |
| Salvo et al. 2022 | Brazil | 284 |  |  |  |  |  |  |  |  | 284 |
| Van Uytsel et al. 2022 | Belgium | 1450 | 948 |  |  |  |  |  | 109 |  | 393 |

## 3.2 Gender

**Table S5 Gender**

| Author | Country | Sample size | Male | Female | Not reported | GNC/ Non binary | Prefer not to say |
| --- | --- | --- | --- | --- | --- | --- | --- |
| Afari et al. 2019 | USA | 88 | 67 | 21 |  |  |  |
| Aherne 2022 | UK | 61 | 10 | 51 |  |  |  |
| Annesi & Eberly 2023 | USA | 121 | 0 | 121 | 0 |  |  |
| Annesi et al. 2016 | USA | 103 |  | 103 |  |  |  |
| Annesi et al. 2019 | USA | 152 |  | 152 |  |  |  |
| Bacon et al. 2005 | USA | 78 | 0 | 78 |  |  |  |
| Berman et al. 2022 | USA | 19 |  | 19 |  |  |  |
| Braden et al. 2022 | USA | 39 | 1 | 38 |  |  |  |
| Carbine et al. 2021 | USA | 100 |  | 53 | 47 |  |  |
| Carpenter et al. 2019 | USA | 75 |  | 69 | 6 |  |  |
| Chung et al. 2016 | USA | 22 | 0 | 22 |  |  |  |
| Daubenmier et al. 2016 | USA | 194 |  | 160 | 34 |  |  |
| Fang et al. 2023 | Taiwan | 20 |  | 15 | 5 |  |  |
| Forman et al. 2013 | USA | 128 |  |  | 128 |  |  |
| Frayn et al. 2020 | Switzerland | 32 | 4 | 28 |  |  |  |
| Genis et al. 2022 | Turkey | 35 | 3 | 32 | 0 |  |  |
| Goldbacher et al. 2016 | USA | 79 | 4 | 75 |  |  |  |
| Hanson et al. 2019 | UK | 53 | 16 | 37 |  |  |  |
| Hanson et al. 2022 | UK | 289 |  | 215 | 74 |  |  |
| Hawkins et al. 2021 | USA | 48 | 8 | 40 |  |  |  |
| Hepdurgan et al. 2020 | Turkey | 51 | 10 | 41 |  |  |  |
| Hunot-Alexander et al. 2021 | UK | 53 | 4 | 49 |  |  |  |
| Kearney et al. 2012 | USA | 48 | 42 | 6 |  |  |  |
| Keränen et al. 2009 | Finland | 82 | 59 | 23 |  |  |  |
| Kidd et al. 2013 | USA | 12 |  | 12 |  |  |  |
| Kim et al 2021 | Korea | 583 | 224 | 359 |  |  |  |
| Lillis et al. 2016 | USA | 162 | 24 | 138 |  |  |  |
| Malkinah-Pykh 2012 | Russia | 104 | 32 | 72 |  |  |  |
| Manchón et al. 2022 | Spain | 23 |  | 9 | 14 |  |  |
| Manzoni et al. 2009 | Italy | 40 |  | 40 |  |  |  |
| Mason et al. 2018 | USA | 104 |  | 104 |  |  |  |
| Mohseni et al. 2022 | Netherlands | 96 |  | 73 | 23 |  |  |
| Moraes et al 2021 | Brazil | 64 |  |  | 64 |  |  |
| Mueller et al. 2022 | UK | 388 | 84 | 303 |  |  | 1 |
| Niemeier et al. 2012 | USA | 21 | 2 | 19 |  |  |  |
| Paans et al. 2020 | Netherlands | 743 | 183 | 560 |  |  |  |
| Palmeira et al. 2017 | Portugal | 27 |  | 27 |  |  |  |
| Paul et al. 2022 | Netherlands | 130 |  | 95 | 35 |  |  |
| Reiger et al. 2017 | Australia | 201 |  | 148 | 53 |  |  |
| Roosen et al. 2019 | Netherlands | 35 | 5 | 30 |  |  |  |
| Salvo et al. 2021 | Brazil | 20 |  | 20 |  |  |  |
| Salvo et al. 2022 | Brazil | 284 | 0 | 284 |  |  |  |
| Sampaio et al. 2021 | Brazil | 55 |  | 55 |  |  |  |
| Spadaro et al. 2017 | USA | 46 | 6 | 40 |  |  |  |
| Tham & Chong 2020 | Australia | 120 | 51 | 69 |  |  |  |
| Thomas et al. 2019 | USA | 51 |  | 51 |  |  |  |
| Van Uytsel et al. 2022 | Belgium | 1450 | 0 | 1450 |  |  |  |
|  |  |  |  |  |  |  |  |

# 4.0 Risk of Bias

## 4.1 RoB2

**Table S6 Risk of Bias of RCTs using RoB2**

| RoB2 Tool | | | | | | |
| --- | --- | --- | --- | --- | --- | --- |
| Author | D1 | D2 | D3 | D4 | D5 | Overall |
| Afari et al. 2019 | Low | Some | Low | Some | Low | Low |
| Ahern 2023 | Low | Some | Low | Low | Low | Low |
| Annesi et al. 2016 | Unlcear | Some | Low | Some | Low | Some |
| Annesi et al. 2019 | Low | Low | Low | Low | Low | Low |
| Bacon et al. 2005 | Low | Some | High | Some | Low | High |
| Berman et al. 2022 | Low | Some | Low | Low | Low | Some |
| Carbine et al. 2021 | Low | Low | Low | Low | Low | Low |
| Carpenter et al. 2019 | Low | Low | Low | Low | Low | Low |
| Daubenmier et al. 2016 | Low | Some | Low | Some | Low | Some |
| Fang et al. 2023 | Low | Some | Low | Low | Low | Some |
| Forman et al. 2013 | Low | Some | Some | Low | Low | Low |
| Goldbacher et al. 2016 | Low | Low | Low | Low | Low | Low |
| Hepdurgan et al. 2020 | Low | Some | Some | Low | Low | Some |
| Keränen et al. 2009 | Low | Low | High | Low | Some | High |
| Kim et al. 2021 | Low | Some | Some | Some | Low | Some |
| Lillis et al. 2016 | Low | Low | Low | Low | Low | Low |
| Malkina-Pykh 2012 | Some | Some | Some | Low | Low | Some |
| Manzoni et al. 2009 | Some | Some | High | Low | Low | High |
| Moraes et al. 2021 | Low | Some | High | Some | Low | High |
| Mueller et al. 2023 | Low | Low | Low | Low | Low | Low |
| Paans et al. 2020 | Low | Low | Low | Low | Low | Low |
| Palmeira et al. 2017 | Low | Low | Low | Low | Low | Low |
| Paul et al. 2022 | Low | Low | Low | Low | Low | Low |
| Rieger et al. 2017 | Low | Low | Low | Low | Low | Low |
| Salvo et al. 2022 | Low | Low | Some | Low | Low | Some |
| Sampaio et al. 2021 | Low | Low | Low | Low | Low | Low |
| Spadaro et al. 2017 | Low | Low | Low | Low | Low | Low |
| Thomas et al. 2019 | Low | Low | Low | Low | Low | Low |
| Van Uytsel et al. 2022 | Some | Low | Some | Some | Low | Some |

## 4.2 ROBINS-I

**Table S7 ROBINS-I assessment of experimental studies**

| ROBINS-1 tool to assess bias in non randomized studies | | | | | | | | |
| --- | --- | --- | --- | --- | --- | --- | --- | --- |
| Author | D1 | D2 | D3 | D4 | D5 | D6 | D7 | Overall |
| Anessi & Eberly (2023) | Moderate | Low | Low | Low | Low | Low | Moderate | Moderate |
| Braden et al. (2022) | Moderate | Low | Low | Low | Low | Low | Low | Moderate |
| Chung et al. (2016) | Moderate | Low | Low | Low | Moderate | Low | Modeerate | Moderate |
| Frayn et al. (2020) | Moderate | Low | Low | Low | Low | Low | Low | Moderate |
| Genis et al.(2022) | Moderate | Low | Low | Low | Moderate | Low | Moderate | Moderate |
| Hanson et al. (2018) | Moderate | Low | Moderate | Moderate | Moderate | Low | Low | Serious |
| Hanson et al. (2022) | Moderate | Moderate | Low | Moderate | Moderate | Low | Low | Moderate |
| Hawkins et al. (2021) | Moderate | Low | Low | Low | Low | Low | Low | Moderate |
| Hunot-Alexander et al. (2021) | Moderate | Low | Low | Low | Moderate | Moderate | Moderate | Moderate |
| Kearney et al. (2012) | Moderate | Low | Low | Moderate | Low | Low | Low | Moderate |
| Kidd et al. (2013) | Moderate | Low | Low | Moderate | Moderate | Moderate | Low | Moderate |
| Manchón et al. (2022) | Moderate | Low | Low | Moderate | High | Moderate | Low | Serious |
| Mason et al. (2022) | Moderate | Low | Low | Moderate | Low | Moderate | Low | Moderate |
| Mohseni et al. (2022) | Moderate | Low | Low | Low | Moderate | Low | Low | Moderate |
| Niemeier et al. (2012) | Moderate | Low | Low | Low | Low | Low | Low | Moderate |
| Roosen et al. (2012) | Moderate | Low | Low | Low | Low | Low | Low | Moderate |
| Salvo et al. (2022) | Moderate | Moderate | Low | Moderate | Moderate | Moderate | Low | Serious |
| Tham & Chong (2020) | Moderate | Low | Low | Low | Low | Low | Moderate | Moderate |

# 5.0 Behaviour Change Techniques

**Table S8 Individual BCTs and impact on weight (Kg) in intervention groups (pre-post)**

| BCT | estimate | conf.low | conf.high | N_stud |
| --- | --- | --- | --- | --- |
| **1.3 Goal setting outcome** | -8.4431032 | -10.463919 | -6.4222874 | 2 |
| **13.3 Incompatible beliefs** | -8.4431032 | -10.463919 | -6.4222874 | 2 |
| **9.3 Imagining future** | -7.92 | -10.846271 | -4.9937289 | 1 |
| **7.5 remove aversive stimulus** | -7.5314001 | -16.282636 | 1.21983625 | 2 |
| **2.2 Feedback on behaviour** | -7.0386349 | -10.683927 | -3.3933424 | 2 |
| **9.2 Pros and cons** | -6.7532617 | -8.7845048 | -4.7220187 | 3 |
| **1.7 Review outcome goals** | -6.6298662 | -8.8447641 | -4.4149683 | 2 |
| **10.1 Material Incentive (behaviour)** | -6 | -9.0306514 | -2.9693486 | 1 |
| **13.1 Self as role model** | -5.8483054 | -10.218974 | -1.4776365 | 3 |
| **9.1 Commitment** | -5.60593 | -10.799008 | -0.4128519 | 4 |
| **13.4 Valued Self identity** | -5.5725587 | -9.0054789 | -2.1396384 | 6 |
| **1.2 Problem Solving** | -5.4505323 | -7.2353148 | -3.6657499 | 20 |
| **12.4 Distraction** | -5.4206483 | -9.3192055 | -1.522091 | 3 |
| **5.6 Information about emotional consequences** | -5.3106624 | -8.2759703 | -2.3453545 | 8 |
| **3.3 Social support unspecified** | -5.2726232 | -7.258827 | -3.2864193 | 10 |
| **1.1 Goal setting (behaviour)** | -5.1609287 | -6.8258049 | -3.4960524 | 21 |
| **15.3 Focus on past success** | -5.0956696 | -8.0189058 | -2.1724334 | 4 |
| **2.1 Self-monitoring of behaviour** | -4.9577646 | -6.6517885 | -3.2637406 | 16 |
| **9.1 Credible source** | -4.8172786 | -6.5233656 | -3.1111915 | 16 |
| **13.2 Framing/reframing** | -4.5641929 | -7.0455217 | -2.0828642 | 15 |
| **8.1 Practice/ rehearsal** | -4.4831513 | -6.2184507 | -2.7478519 | 22 |
| **1.4 Action planning** | -4.3792262 | -5.9166096 | -2.8418428 | 13 |
| **5.4 Monitoring of emotional consequences** | -4.3345158 | -8.013174 | -0.6558576 | 6 |
| **4.1 Instructions on how to perform** | -4.3060721 | -5.6734182 | -2.9387259 | 30 |
| **5.1 information about health consequences** | -4.2456551 | -6.5644827 | -1.9268275 | 9 |
| **6.2 Social comparison** | -4.2332998 | -7.0629195 | -1.40368 | 3 |
| **12.1 Restructure physical environment** | -4.187087 | -6.9729523 | -1.4012218 | 3 |
| **2.4 Self monitoring of outcomes of behaviour** | -3.9307893 | -5.6493591 | -2.2122195 | 6 |
| **1.8 Behavioural contract** | -3.8478875 | -8.2434265 | 0.54765152 | 2 |
| **1.6 Discrepancy** | -3.8478875 | -8.2434265 | 0.54765152 | 2 |
| **8.7 Graded tasks** | -3.8478875 | -8.2434265 | 0.54765152 | 2 |
| **7.1 Prompts/ cues** | -3.8478875 | -8.2434265 | 0.54765152 | 2 |
| **6.1 Demonstration of behaviour** | -3.8459398 | -5.3482686 | -2.343611 | 20 |
| **11.2 Reduce negative emotions** | -3.7778854 | -5.1542418 | -2.401529 | 24 |
| **12.5 Adding objects to environment** | -3.298698 | -4.9992621 | -1.5981338 | 3 |
| **4.4 Behavioural experiments** | -2.2664361 | -4.969058 | 0.43618583 | 3 |
| **8.3 Habit formation** | -1.906434 | -4.3057236 | 0.49285552 | 4 |
| **10.6 Nonspecific incentive** | 0.45 | -4.1801052 | 5.08010519 | 1 |
| **3.3 Social support (emotional)** | 0.7 | -3.9499196 | 5.34991958 | 1 |

**Table S9 Individual BCTs and impact on SMD in EE score in intervention groups (pre-post)**

| BCT | estimate | conf.low | conf.high | Number of Studies |
| --- | --- | --- | --- | --- |
| 13.5 Identity with changed behaviour | -1.4947 | -1.9989 | -0.9906 | 1 |
| 13.3 Incompatible eliefs | -1.4601334 | -1.8215368 | -1.09873 | 3 |
| 1.3 Goal setting (outcome) | -1.4575216 | -2.0122128 | -0.9028305 | 2 |
| 3.3 Social support (emotional) | -1.4215428 | -2.059089 | -0.7839966 | 1 |
| 1.7 Review outcome goals | -1.2179167 | -1.4284702 | -1.0073631 | 2 |
| 9.3 Imagining future | -1.1865373 | -1.4449784 | -0.9280961 | 1 |
| 7.5 Remove stimulus | -1.1840246 | -1.4699985 | -0.8980507 | 4 |
| 2.2 Feedback on behaviour | -1.1186669 | -1.5278389 | -0.7094948 | 4 |
| 9.2 Pros cons | -1.093338 | -1.7994083 | -0.3872676 | 3 |
| 5.4 Monitoring emotional consequences | -1.0358438 | -1.5193303 | -0.5523572 | 7 |
| 12.4 Distraction | -0.9802054 | -1.5206755 | -0.4397354 | 4 |
| 13.2 Framing reframing | -0.9730767 | -1.1636882 | -0.7824651 | 22 |
| 4.2 Information about antecedents | -0.964 | -1.2659 | -0.6621 | 7 |
| 5.6 Information emotional consequences | -0.9572316 | -1.26441 | -0.6500532 | 12 |
| 13.1 Self role model | -0.9530122 | -1.7454129 | -0.1606115 | 3 |
| 1.2 Problem solving | -0.9472038 | -1.1186668 | -0.7757408 | 27 |
| 1.1 Goal setting | -0.9295813 | -1.1172843 | -0.7418783 | 27 |
| 1.9 Commitment | -0.9271392 | -1.2133306 | -0.6409479 | 10 |
| 2.3 Self monitoring of behaviour | -0.9244861 | -1.1177891 | -0.7311831 | 22 |
| 5.1 Information health consequences | -0.9133323 | -1.4402939 | -0.3863707 | 9 |
| 3.1 Social support (unspecified) | -0.902946 | -1.2484843 | -0.5574076 | 12 |
| 2.1 Monitoring of behaviour by others | -0.8988 | -1.5084 | -0.2892 | 6 |
| 8.2 Behaviour substitution | -0.8805 | -1.2176 | -0.5434 | 11 |
| 7.1 Prompts cues | -0.8641889 | -1.202017 | -0.5263609 | 5 |
| 13.4 Valued self id | -0.8609888 | -1.1243683 | -0.5976092 | 11 |
| 4.4 Behavioural experiments | -0.8477577 | -1.4520871 | -0.2434284 | 3 |
| 1.6 Discrepancy | -0.8392966 | -1.4482924 | -0.2303008 | 3 |
| 11.2 Reduce negative emotions | -0.8365809 | -1.0621928 | -0.6109689 | 29 |
| 2.5 Monitoring of outcomes of behaviour | -0.8342 | -1.4355 | -0.2329 | 3 |
| 8.1 Practice rehearsal | -0.8237268 | -1.0362342 | -0.6112194 | 25 |
| 4.1 Instructions on behaviour | -0.8173964 | -0.9950571 | -0.6397357 | 37 |
| 1.4 Action Planning | -0.8070829 | -1.1072668 | -0.5068989 | 15 |
| 12.5 Adding objects | -0.7842479 | -1.3546866 | -0.2138091 | 4 |
| 9.1 Credible source | -0.7568214 | -1.0155047 | -0.498138 | 20 |
| 6.1 Demonstrate behaviour | -0.7558591 | -0.9727174 | -0.5390008 | 25 |
| 2.4 Self monitoring of outcomes behaviour | -0.7450568 | -1.1826334 | -0.3074802 | 6 |
| 8.7 Graded tasks | -0.7311009 | -1.013758 | -0.4484439 | 4 |
| 5.3 Information social and environmental | -0.6781 | -1.1504 | -0.2058 | 7 |
| 1.5 Review Behaviour | -0.6706 | -0.9466 | -0.3947 | 11 |
| 12.1 Restructure physical environment | -0.641503 | -0.9457819 | -0.3372242 | 4 |
| 8.3 Habit formation | -0.6369031 | -0.8422552 | -0.4315509 | 4 |
| 10.1 Material incentive (behaviour) | -0.5714021 | -0.8154944 | -0.3273098 | 1 |
| 5.5 Anticipated regret | -0.527 | -0.8179 | -0.236 | 2 |
| 8.4 Habit reversal | -0.527 | -0.8179 | -0.236 | 2 |
| 1.8 Behavioural contract | -0.5269668 | -0.8179043 | -0.2360293 | 2 |
| 2.7 Feedback on outcomes of behaviour | -0.4702 | -1.2093 | 0.2688 | 2 |
| 12.3 Reducing exposure to cues | -0.4517 | -0.7069 | -0.1965 | 1 |
| 6.2 Social comparison (group) | -0.1636152 | -1.3537663 | 1.02653595 | 2 |
| 3.2 Social support (practical) | -0.0998 | -0.215 | 0.0153 | 1 |
| 15.3 Past success | -0.0950181 | -0.6051499 | 0.41511367 | 3 |

# 6.0 Sensitivity analysis**:**

## 6.1 Weight change.

There was no moderation effect of length of intervention in weeks (< 14 weeks vs 14 – 30 weeks vs >30 weeks: X2(2) = 5.59, p = .061). For < 14 weeks (N = 14) the reduction in weight was -2.12 kg [95% CI: -3.16 to -1.08]). For 14 – 30 weeks (N = 14) the reduction in weight was -5.27 kg [95% CI: -7.54 to -3.01]. For >30 weeks (N = 4) the reduction in weight was -5.59 [95% CI: -9.62 to -1.57].

There was no moderation effect by intervention delivery (X2(1) = 1.62, p = .203). For in person delivery (N = 26) the reduction in weight was -4.48 [95% CI: - 6.02 to -2.9]. For non-in person delivery (N = 6) it was -1.90 [95% CI: -3.17 to -0.64].

There was no moderation by intervention format (X2(2) = 3.00, p = .223). For group interventions (N = 20) the reduction in weight was -4.59 [95% CI: -6.10 to -3.07]. For individual interventions (N = 7) the reduction in weight was -2.18 [95% CI: -2.98 to -1.37]. For mixed format (N = 4) the reduction in weight was -5.23 [95% CI: -10.52 to 0.07].

There was no moderaiton by study type (X2(1) = 1.93, p = .165). For RCTs (N = 21) the reduction in weight was -3.42 [95% CI: -4.66 to -2.19]. For quasi-designs (N = 11) the reduction in weight was -5.23 [95% CI: -8.27 to -2.19].

There was no moderation by study quality (X2(1) = 0.09, p = .770). For high quality studies (N = 24) the reduction in weight was -4.22 [95% CI: -5.81 to -2.62]. For moderate quality studies (N = 8) the reduction in weight was -3.77 [95% CI: -6.34 to -1.18].

## 6.2 Emotional Eating

There was no moderation effect of length of intervention in weeks (< 14 weeks vs 14 – 30 weeks vs >30 weeks: X2(2) = 5.28, p = .072). For < 14 weeks (N = 16) the effect sizes was SMD = -0.57 [95% CI: -0.82 to -0.32]). For 14 – 30 weeks (N = 16) the effect size was SMD = -0.95 [95% CI: -1.29 to -0.65]. For >30 weeks (N = 7) the effect size was SMD = -1.01 [95% CI: -1.30 to -0.72].

There was no moderation effect by intervention delivery (X2(1) = 0.08, p = .776). For in person delivery (N = 36) the reduction in emotional eating was SMD = -0.82 [95% CI: -1.02 to -0.63]. For non-in person delivery (N = 6) the effect size was SMD = -0.76 [95% CI: -1.12 to -0.40].

There was no moderation by intervention format (X2(2) = 1.29, p = .524). For group interventions (N = 25) the effect size was SMD = -0.81 [95% CI: -1.05 to -0.60]. For individual interventions (N = 9) the effect size was SMD = -0.69 [95% CI: -1.00 to -0.38]. For mixed format (N = 5) the reduction in weight was -1.03 [95% CI: -1.41 to -0.65].

There was no moderation by study type (X2(1) = 0.42, p = .515). For RCTs (N = 25) the effect size was SMD = -0.76 [95% CI: -0.94 to -0.58]. For quasi-designs (N = 14) the reduction in weight was -0.90 [95% CI: -1.25 to -0.52].

There was no moderation by study quality (X2(1) = 0.06, p = .780). For high quality studies (N = 28) the effect size was SMD = -0.79 [95% CI: -0.98 to -0.60]. For moderate quality studies (N = 11) the effect size was -0.86 [95% CI: -1.25 to -0.47].

## 6.3 Publication bias

**Figure S1: Funnel Plot for weight**


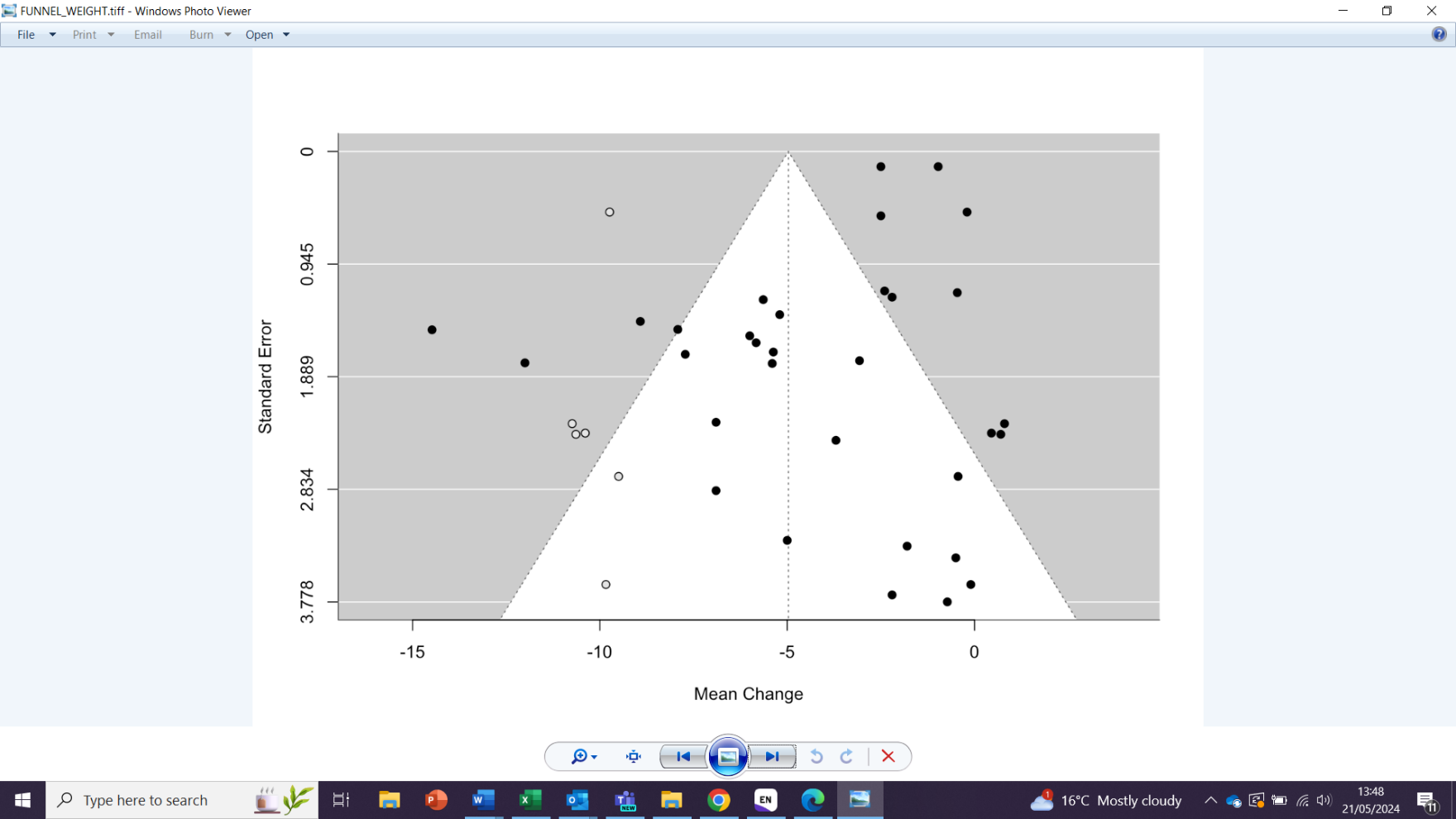


**Figure S2: Funnel Plot for EE**


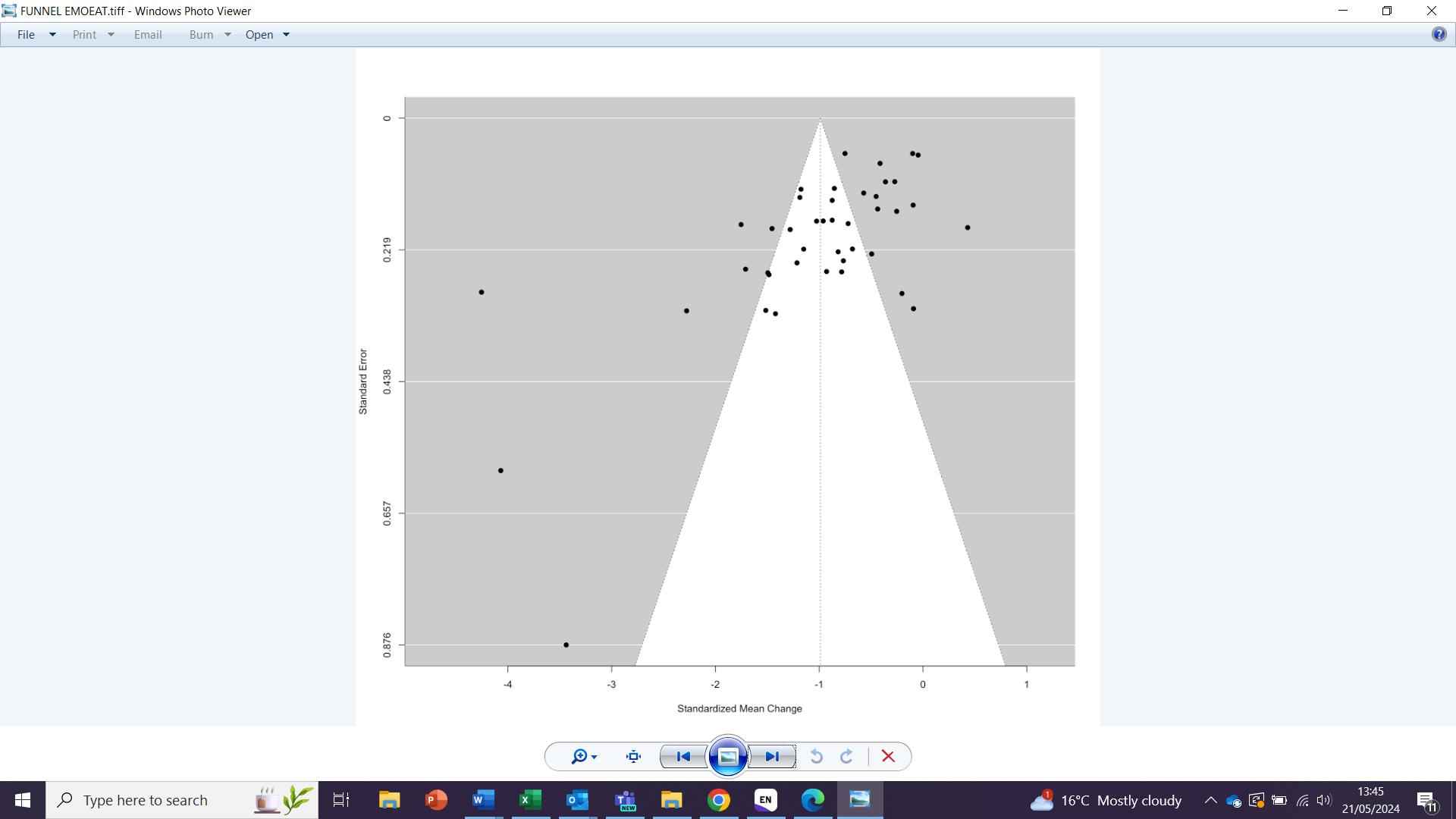


# 7.0 BCT analysis Intervention vs. Control

The primary analysis described in this review is a pre-post analysis combining observational and RCT data, excluding the control group data because of similar overlapping BCTs in both groups. However, we recognise the limitations of observational data, and have therefore run an additional analysis of RCTs, comparing impact of BCTs in intervention groups to impact of BCTs in control groups.

In this additional analysis, we focused exclusively on BCTs that are unique to the intervention group, excluding those that are common to both the intervention and control groups.

This analysis showed, only 1 BCT (review outcome goal) was associated with statistically significant changes in weight (see figure S3). Although not significant, ‘framing/re-framing’, ‘problem solving and ‘review behaviour’ tended towards a relatively higher weight loss compared to other BCTs. This is reflective of the findings in the main analysis which demonstrated that reviewing goals was associated with significant weight loss and changes in EE scores.

Four BCTs were associated with statistically significant reduction in EE scores. relative to control: ‘demonstration of behaviour’, ‘behavioural practice/rehearsal’, ‘reduce negative emotions’ and ‘review behaviour’ (see figure S4). These BCTs were also associated with significant changes in the primary analysis, however the emphasis on demonstrating and practicing of skills is highlighted in this additional analysis.

However, these findings are not included in the main text for several reasons: the need to exclude BCTs common to both interventions may obscure the impact of these BCTs; even when a BCT was present in both the intervention and control groups, it was not necessarily implemented at the same dose. Therefore, this additional analysis risks overlooking the potential impact of the excluded BCTs. While observational findings are limited, we agreed that the pre-post analysis was the most appropriate in this instance.

Furthermore, our GRADE assessment indicated a low level of certainty which is consistent with the observational nature of the BCT analysis. Therefore, this systematic review indicates which BCTs show promise and warrant further testing in high quality RCTs comparing BCT interventions with non BCT/minimal intervention controls.

We have made recommendations to address this in future BCT and EE research (see conclusions and table S9 Conditional Recommendations.

**Figure S3 : Forest plot of BCTs and their impact weight loss relative to control group**


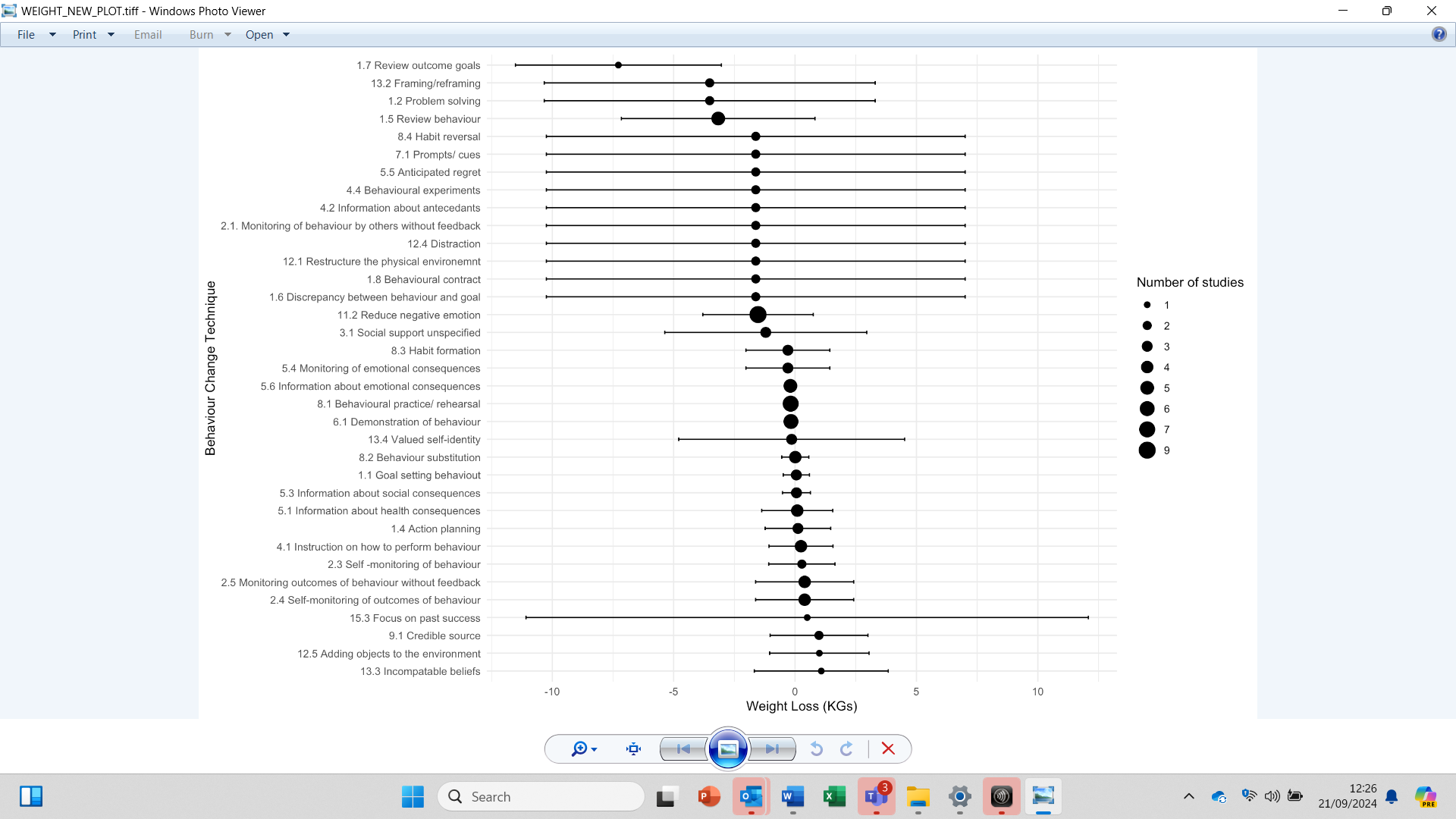


**Figure S4: Forest plot of BCTs and their impact on EE relative to control group**


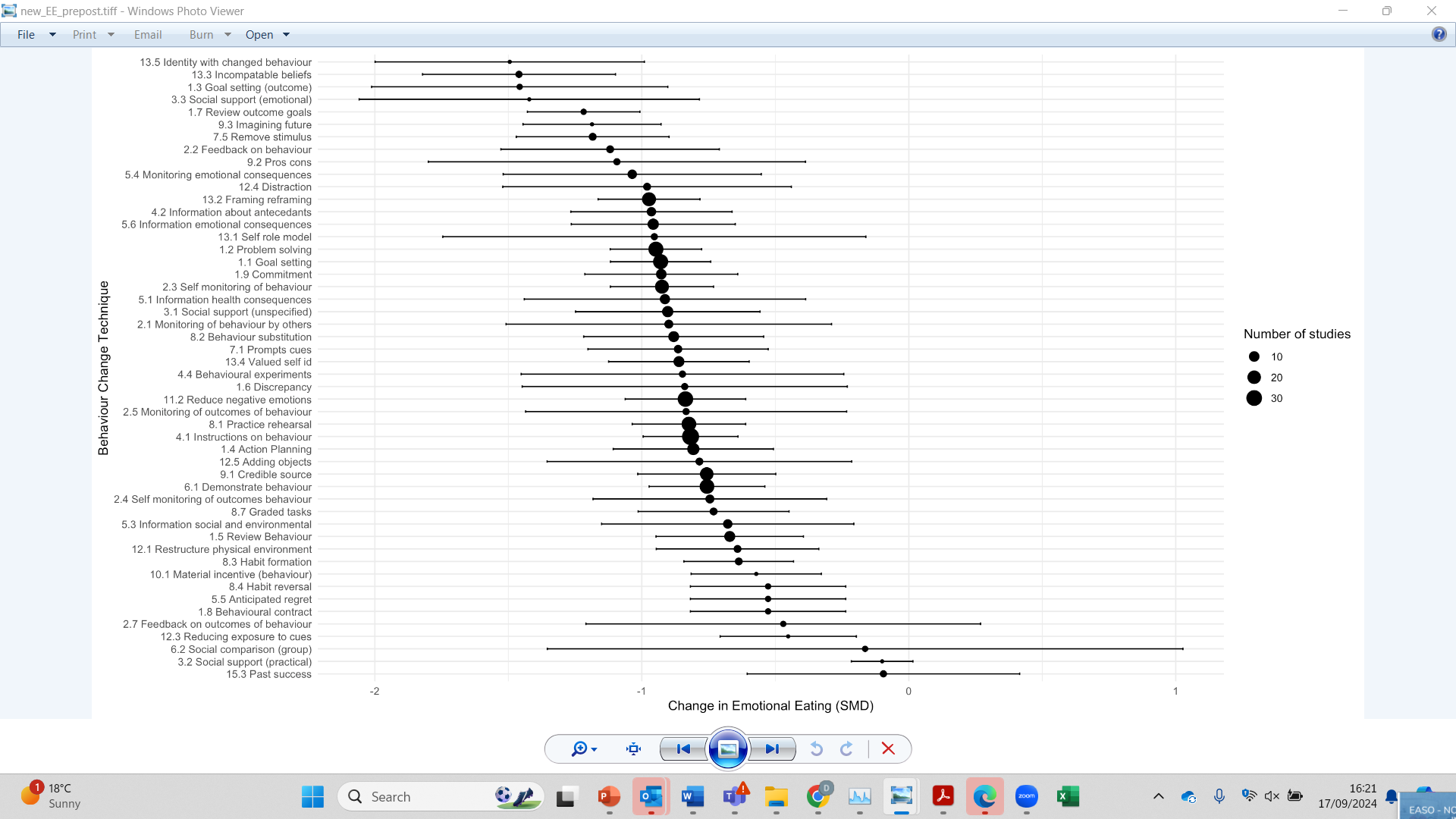


# 8.0 GRADE Assessment

**Table S10 GRADE Assessment of Certainty of Evidence**

| Outcome | Overall impact of interventions on outcome | No. of Participants (Studies) | Quality of Evidence (GRADE) | Comments on GRADE Assessment |
| --- | --- | --- | --- | --- |
| Emotional Eating | -0.99 [95% CI: -0.73 to -1.25],  p < .001, I^2^ = 97%, tau^2^ = 0.68 | 5185 (42) | Low Confidence  ◯⊕◯⊕◯ | **Risk of bias:**  55.7% of participants in EE studies belong to studies assessed as some risk of bias, and 40.5% of participants in EE studies were from studies with low risk of bias, with only 3.8% of participants from studies assessed as high risk of bias. Given given that non RCT studies and studies without a comparator are included in the meta-analysis, risk of bias has been marked down.  **Imprecision:**  The effect estimate for EE suggests that interventions targeting EE achieve a statistically significant reduction in SMD score with a relatively narrow 95% CI. However, what constitutes as a clinically meaningful reduction is unknown. Removal of outliers did not change the direction of the results, and 95% CI remained narrow, although the effect was less pronounced. Therefore, some concern with imprecision is noted but has not been marked down for this domain.  **Inconsistency:** All studies measuring EE demonstrated a reduction in SMD score for EE, with a small number of studies (5) including the line of no effect within the 95% CI. However, Heterogeneity was significant across studies, even after removal of outliers (I^2^ = 93%). Therefore, confidence in the results is marked down in this domain.  **Indirectness:**  Only studies which were consistent with our PICO criteria were included in analysis, therefore the study population and interventions of interest were appropriate and match the population of interest for which any recommendations apply to.  **Publication bias**  Some risk of publication bias detected as Egger’s test was statistically significant (z = 5.25, p < .001), therefore is marked down for this domain. |
| Weight | -4.09 kg [95% CI: -2.76 to -5.43 kgs],  p < .001, I^2^ = 96%, tau2 = 11.06 | 4062 (32) | ◯◯◯⊕⊕  Low confidence | **Risk of bias:**  57.5% of participants are from studies with some risk of bias, 37.4% of participants are from studies of low risk of bias and 5.1% of participants are from studies deemed as high risk of bias.. Additionally, given risk of seletion bias associated with non-randomised studies this domain has been marked down.  **Imprecision:**  The effect estimate for weight suggests that taken together, interventions achieved a statistically significant reduction in weight. Leave-one-out analyses did not lead to any substantial deviations from the overall pooled effect and all models remained significant. However, 22 studies had low precision with 95% CI exceeding 5kg. Therefore, this domain is marked down.  **Inconsistency:** 14 out of 31 studies which measured impact of interventions on weight, included the line of no impact within their 95% CI. Furthermore, heterogeneity was significant for this outcome at I^2^ = 97% Therefore, confidence in the results is marked down in this domain.  **Indirectness:**  Only studies which were consistent with our PICO criteria were included in analysis, therefore the study population and interventions of interest were appropriate and match the population of interest for which any recommendations apply to.  **Publication bias**  Low risk of publication bias detected as Egger’s test was not statistically significant (Z = .37, p = .714) and Leave-one-out analyses did not lead to any substantial deviations from the overall pooled effect; all models remained significant. Therefore there was not enough evidence to mark down based on publication bias for weight. |

# 9.0 Recommendations

**Table S11 Conditional Recommendations**

| Implications | Conditional recommendations |
| --- | --- |
| For Patients | Patients should have the opportunity to access psychological support within weight management services. |
| For clinicians | We would suggest that clinicians working within weight management consider screening patients accessing the services for presence of EE. |
| For policymakers | Consider developing treatment pathways within weight management services with psychological support for EE. Psychological interventions targeting EE should consider inclusion of BCTs highlighted in this review, with an emphasis on identity, values, and self-compassion. |
| For further research | Further testing and implementation of BCTs highlighted in this review is needed, using high quality RCTs comparing BCT intervention group to non-BCT or minimal intervention controls. |
